# Bathing Effects of Various Seawaters on Allergic (Atopic) Dermatitis-Like Skin Lesions Induced by 2,4-Dinitrochlorobenzene in Hairless Mice

**DOI:** 10.1155/2015/179185

**Published:** 2015-06-16

**Authors:** Choong Gon Kim, Meehye Kang, Youn-Ho Lee, Won Gi Min, Yong Hwan Kim, Su Jin Kang, Chang Hyun Song, Soo Jin Park, Ji Ha Park, Chang Hyun Han, Young Joon Lee, Sae Kwang Ku

**Affiliations:** ^1^Biological Oceanography & Marine Biology Division, KIOST, Ansan 426-744, Republic of Korea; ^2^Dokdo Research Center, East Sea Research Institute, KIOST, Ansan 426-744, Republic of Korea; ^3^Gyeongbuk Institute for Marine Bio-Industry, Gyeongbuk 787-813, Republic of Korea; ^4^The Medical Research Center for Globalization of Herbal Formulation, Daegu Haany University, Gyeongsan 712-715, Republic of Korea; ^5^Department of Preventive Medicine, College of Korean Medicine, Daegu Haany University, Gyeongsan 712-715, Republic of Korea; ^6^Department of Anatomy and Histology, College of Korean Medicine, Daegu Haany University, Gyeongsan 712-715, Republic of Korea; ^7^Department of Herbology, College of Korean Medicine, Daegu Haany University, Gyeongsan 712-715, Republic of Korea; ^8^Department of Medical History & Literature Group, Korea Institute of Oriental Medicine, Daejeon 305-811, Republic of Korea

## Abstract

We evaluated the preventive effects of four types of seawater collected in Republic of Korea on hairless mice with 2,4-dinitrochlorobenzene- (DNCB-) induced allergic/atopic dermatitis (AD). The anti-inflammatory effects were evaluated by measuring tumor necrosis factor- (TNF-) *α* and interleukins (ILs). Glutathione (GSH), malondialdehyde (MDA), superoxide anion, and inducible nitric oxide synthase (iNOS) were measured to evaluate the antioxidant effects. Caspase-3 and poly (ADP-ribose) polymerase (PARP) were observed to measure the antiapoptotic effects; matrix metalloproteinase- (MMP-) 9 levels were also evaluated. Mice with AD had markedly higher clinical skin severity scores and scratching behaviors; higher TNF-*α* and ILs (1*β*, 10, 4, 5, and 13) levels; higher MDA, superoxide anion, caspase-3, PARP, and MMP-9 levels; and greater iNOS activity. However, the severity of AD was significantly decreased by bathing in seawaters, but it did not influence the dermal collagen depositions and skin tissue antioxidant defense systems. These results suggest that bathing in all four seawaters has protective effects against DNCB-induced AD through their favorable systemic and local immunomodulatory effects, active cytoprotective antiapoptotic effects, inhibitory effects of MMP activity and anti-inflammatory and antioxidative effects.

## 1. Introduction

Allergic/atopic dermatitis (AD), a chronic inflammatory skin disease associated with cutaneous hyperreactivity, affects approximately 3% of infants, 10–20% of children, and 1–3% of adults worldwide [1]. Patients with AD develop extremely itchy skin followed by severe scratching behavior that induces the production of proinflammatory cytokines [2]. This in turn activates immune cells and initiates an inflammatory cycle of AD accompanied by erythema, keratosis, and scaling [3]. All of these symptoms are the consequence of an imbalanced immune response to various allergens [4]. Another defining characteristic of the allergic immune system is the capacity to generate elevated immunoglobulin E (IgE) antibodies and type 2 helper T cells (Th2), which are critical for IgE synthesis [5]. The IgE level is associated with the severity of AD, and the abnormal skin barrier in patients with AD, a key feature of the disease, contributes to the increased severity [6]. The functions of IgE in allergic inflammation suggest that IgE and IgE-mediated activation of mast cells and eosinophils contribute to AD. IgE can sensitize mast cells in the skin, culminating in the production of inflammatory mediators such as cytokines (interleukin- (IL-) 4, IL-5, IL-13, and tumor necrosis factor- (TNF)-*α*) when cell-bound IgE is cross-linked by allergens. The cytokines IL-4 and IL-13, which are released by mast cells, contribute to the Th2 response. TNF-*α*, which is produced by macrophages, also plays an important role in the acute phase of AD [7]. Moreover, oxidative stress is involved in the pathogenesis of AD [4].

Adrenocorticosteroids and antihistamine agents have shown favorable ameliorating effects for the treatment of AD, but they have also shown serious side effects [8]. Accordingly, researchers have explored alternative therapies and natural products in a vigorous attempt to ameliorate AD [4, 6]. Hydrotherapy is among these alternative approaches [9] and may be used as a complementary therapy. Indeed, several studies have tested different types of water baths and reported beneficial effects on dermatological disorders [10–13]. Even when the composition of the water baths differs, each has somewhat unique characteristics [14]. Hydrotherapy can reportedly modulate lymphocyte proliferation and cytokine production [14], and some compositions of mineral waters have shown favorable antioxidant effects [15–18]. Moreover, various types of seawaters have shown favorable effects on different types of dermatitis [19–21].

In the present study, we evaluated the anti-AD effects of various types of seawaters collected in Republic of Korea, namely, west surface seawater (WSSW) collected from Wepo-ri, Ganghwa-do; west saline groundwater (WSGW) collected from Yonggungoncheon, Seokmo-do; east surface seawater (ESSW) collected from Nagok-ri, Uljin; and east saline groundwater (ESGW) collected from Hoojeong-ri, Uljin, (Table 1). The anti-AD effects of these seawaters were assessed using a hairless mouse model of 2,4-dinitrochlorobenzene- (DNCB-) induced AD [22].

## 2. Methods

### 2.1. Animals and Husbandry

Total one-hundred twenty-six 6-week female SKH-1 hairless mice (OrientBio, Seongnam, Republic of Korea) were prepared, and seven groups of eight mice each were selected based on the body weights at 5 weeks after DNCB sensitization based on the body weights, clinical skin severity scores, and scratching behaviors. Animals were allocated four per polycarbonate cage in a temperature (20–25°C) and humidity (50–55%) controlled room. Light : dark cycle was 12 hr : 12 hr, and standard rodent chow (Samyang, Seoul, Republic of Korea) and water were supplied free to access. All laboratory animals were treated according to the national regulations of the usage and welfare of laboratory animals and approved by the Institutional Animal Care and Use Committee in Daegu Haany University (Gyeongsan, Gyeongbuk, Republic of Korea).

### 2.2. Preparation of Seawaters and DEXA

Colorless clear solutions of WSSW, WSGW, and ESSW and light yellowish solution of ESGW were collected around Wepo-ri (Ganghwa-do, Republic of Korea), Yonggungoncheon (Seokmo-do, Republic of Korea), Nagok-ri (Uljin, Republic of Korea), and Hoojeong-ri (Uljin, Republic of Korea), respectively, and used after filtration with pore size 1.2 *μ*m GF/C Grass Microfiber Filter (Korea Filter Paper Co., Ltd., Seoul, Republic of Korea) except for WSGW, which was not filtered before being used in this experiment. Salinity and mineral compositions of individual seawaters were listed in Table 1. White powders of water-soluble DEXA (Sigma-Aldrich, St. Louis, MO, USA) was obtained and used in the present study as a potent reference agent. All test materials tested in this experiment were stored at 4°C in a refrigerator to protect from light and humidity until being used. Seawaters were warmed around 37°C, at least 30 min before bathing.

### 2.3. Inducement of AD

**Figure 1:**
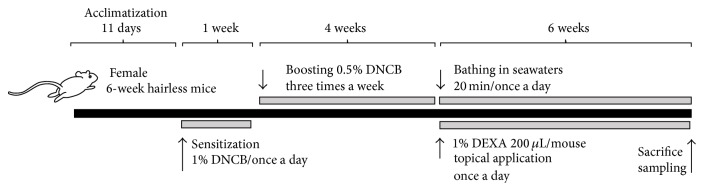
Experimental designs used in this study. AD-like dermatitis was induced by sensitization of 1% DNCB once a day for 1 week and boosted by 0.5% DNCB, three times a week for 28 days. Each of eight mice per group was freely bathing 20 min/day. 1% DEXA was topically applied on the dorsal back skins once a day for 6 weeks from 5 weeks after DNCB sensitization. Intact and DNCB control mice were bathing on the distilled water, instead of seawaters to provide the same swimming stresses. Six weeks of bathing periods in this study were selected according to previous bathing effects of mineral-rich water.

AD-like dermatitis was induced by sensitization of 1% DNCB (dissolved in a 3 : 1 mixture of acetone and olive oil) once a day for 1 week and boosted by 0.5% DNCB, three times a week for 28 days according to established previous methods [4, 6] with some modifications. DNCB solutions were topically applied on the dorsal back skins in a volume of 200 *μ*L/mouse. In intact control mice, only vehicle (3 : 1 mixtures of acetone and olive oil) was topically applied, instead of DNCB solutions in this experiment (Figure 1).

### 2.4. Bathing and Topical Application of DEXA

Each of eight mice per group was freely bathing on the mouse polycarbonate cages (200 × 260 × 130 mm; DJ-101, Daejong Instrument Ind. Co., Seoul, Republic of Korea) containing about 1,900 mL of warm seawaters around 37°C and fasting plates being 4 cm deep for 20 min/day. Water-soluble DEXA was dissolved in distilled water as 1% solution and topically applied on the dorsal back skins as 200 *μ*L/mouse once a day for 6 weeks from 5 weeks after DNCB sensitization. Intact and DNCB control mice were bathing on the distilled water, instead of seawaters in this experiment, to provide the same swimming stresses. Seawaters and 1% DEXA solutions were warmed around 37°C, at least 30 min before bathing to avoid cooling irritations. Six weeks of bathing periods in this study were selected according to previous bathing effects of mineral-rich water [23] (Table 1, Figure 1).

### 2.5. Changes in Body Weights

Changes of body weight were measured at once a week from 1 day before initial DNCB sensitization to end of 6 weeks of continuous bathing in the 4 different types of seawaters or topical application of 1% DEXA using an automatic electronic balance (Precisa Instrument, Dietikon, Switzerland). To reduce the individual differences, the total body weight gains during 11 weeks of the whole experimental periods and body weight gains during 6 weeks of bathing or topical application of 1% DEXA were calculated as follows, respectively:
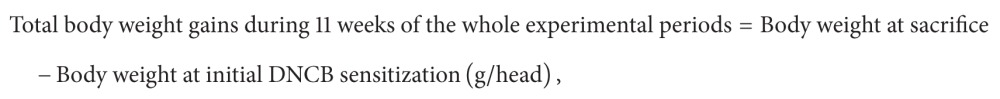
(1)

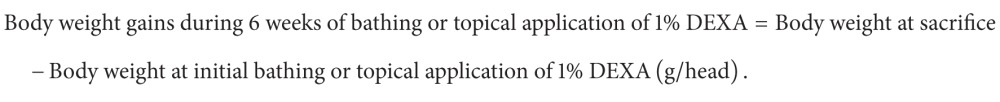
(2)


### 2.6. Evaluation of Clinical Skin Severity Scores

Five signs of skin lesions, (1) pruritus/itching, (2) erythema/hemorrhage, (3) edema, (4) excoriation/erosion, and (5) scaling/dryness, were graded as follows: 0 (no symptoms), 1 (mild), 2 (moderate), and 3 (severe), and totalized scores (max = 15) were regarded as clinical skin severity score based on the previous report [4] with some modifications. Scoring was conducted, at 35, 38, 42, 49, 56, 63, 70, and 77 days after first DNCB sensitization, respectively.

### 2.7. Evaluation of Scratching Behavior

Each mouse was placed individually in a routine polycarbonate mouse cage and their behavior was monitored for 30 min, at 35, 38, 42, 49, 56, 63, 70, and 77 days after first DNCB sensitization, respectively. Scratching of the rostral back and biting of the caudal back were observed; scratching movements by the hind paw were defined as a scratching bout that ended when the mice either licked their hind paw or placed their hind paw back on the floor, and a series of one or more biting movements were counted as one episode that ended when the mouse returned to the straight-forward position [4].

### 2.8. Serum Total IgE Level Measurement

At sacrifice, about 1 mL of venous blood was collected from vena cava under anesthesia with 2 to 3% isoflurane (Hana Pharm. Co., Hwaseong, Republic of Korea) in the mixture of 70% N_2_O and 28.5% O_2_, and serum was separated by centrifuging at 15,000 rpm for 10 min under 4°C, using clotting activated serum tube. Total IgE levels in serum were determined by sandwich ELISA using the mouse IgE ELISA set (BD Biosciences, San Diego, CA, USA) according to previous methods [4, 6]. Briefly, plates were coated with capture antibody in ELISA coating buffer and incubated overnight at 4°C. Plates were washed with PBS-Tween 20 (0.05%) and subsequently blocked (10% FBS in PBS) for 1 hr at 20°C. Serial dilutions of standard antigen or sample in dilution buffer (10% FBS in PBS) were added to the plates and plates were incubated for 2 hrs at 20°C. After washing, biotin-conjugated anti-mouse IgE and streptavidin-horseradish peroxidase conjugate were added to the plates and plates were incubated for 1 hr at 20°C. Finally, tetramethylbenzidine substrate solution was added to the plates and after 15 min of incubation in the dark, a 2NH_2_SO_4_ solution was added to stop the reaction. Optical densities were measured at 450 nm on an automated ELISA reader (Tecan; Männedorf, Switzerland).

### 2.9. Lymphatic Organ Weight Measurements

At sacrifice, 24 hrs after end of last 42th bathing, the spleen and left submandibular LN in each mouse were collected after eliminations of the surrounding connective tissues, muscles, and any debris. Individual weights of lymphatic organs were measured at g levels regarding absolute wet weights. To reduce the individual body weight differences, the relative weight (% as body weights) was calculated using body weight at sacrifice and absolute organ weights as follows according to our previously established methods [24]:

(3) Relative organ weight % versus body weights=Absolute organ weightBody weight at sacrifice×100.

### 2.10. Splenic Cytokine Content Measurements

Splenic concentrations of TNF-*α*, IL-1*β*, and IL-10 were measured by ELISA using commercially available kits, mouse TNF-*α* ELISA kit (BD Biosciences/Pharmingen, San Jose, CA, USA), mouse IL-1*β* ELISA kit (Genzyme, Westborough, MA, USA), and mouse IL-10 ELISA kit (Genzyme, Westborough, MA, USA), respectively, as previously described [25]. Approximately 10–15 mg of tissue samples were homogenized in a tissue grinder containing 1 mL of lysis buffer (PBS containing 2 mM PMSF and 1 mg/mL of aprotinin, leupeptin, and pepstatin A) as described by Clark et al. [26]. Analysis was performed with 100 mL of standard (diluted in lysis buffer) or 10, 50, or 100 mL of tissue homogenate. Each sample was run in duplicate, and a portion of the sample was analyzed for protein. Data are expressed as pg/mg of protein. For each assay a standard curve was generated and, based on replicates of the measured absorbance, demonstrated an average coefficient of variance of <10%.

### 2.11. Quantitative RT-PCR

Total RNA was extracted using Trizol reagent (Invitrogen, Carlsbad, CA, USA), according to the method described in previous studies [6, 27]. The RNA concentrations and quality were determined by CFX96 Real-Time System (Bio-Rad, Hercules, CA, USA). To remove contaminating DNA, samples were treated with recombinant DNase I (DNA-free; Ambion, Austin, TX, USA). RNA was reverse transcribed using the reagent High-Capacity cDNA Reverse Transcription Kit (Applied Biosystems, Foster City, CA, USA) according to the manufacturer's instructions. The expression of GAPDH mRNA was used as a control for tissue integrity in all samples.

### 2.12. GSH Assay

Cutaneous GSH levels were determined using a fluorescence assay as previously described [28]. Firstly, the skin (1 : 3, w/w dilution) was homogenized in 100 mM NaH_2_PO_4_ (pH 8.0; Sigma-Aldrich, St. Louis, MO, USA) containing 5 mM EDTA (buffer 1). After that, homogenates were treated with 30% trichloroacetic acid (Sigma-Aldrich, St. Louis, MO, USA) and centrifuged twice (at 1,940 ×g for 6 min and at 485 ×g for 10 min) and the fluorescence of the resulting supernatant was measured in a fluorescence spectrophotometer (RF-5301PC; Shimadzu Corp., Tokyo, Japan). Briefly, 100 *μ*L of the supernatant was mixed with 1 mL of buffer 1 and 100 *μ*L of o-phthalaldehyde (1 mg/mL in methanol; Sigma-Aldrich, St. Louis, MO, USA). The fluorescence was determined after 15 min (*k*_exc_ = 350 nm; *k*_em_ = 420 nm). The standard curve was prepared with different concentrations of GSH (0.0–75.0 *μ*M). Protein levels in the skin homogenates were measured using the method of Lowry et al. [29]. Results are presented as *μ*M of GSH/mg of protein.

### 2.13. Lipid Peroxidation

Firstly, the protein content of homogenate (10 mg/mL in 1.15% KCl) was measured using the Lowry et al. method [29]. Thiobarbituric acid reactive substances (TBARS) measurement was used to evaluate lipid peroxidation as previously described [30]. For this assay, trichloroacetic acid (10%; Sigma-Aldrich, St. Louis, MO, USA) was added to the homogenate to precipitate proteins. This mixture was then centrifuged (3 min, 1,000 ×g). The protein-free sample was extracted and thiobarbituric acid (0.67%) was added. The mixture was kept in water bath at 100°C for 15 min. MDA, an intermediate product of lipoperoxidation, was determined by difference between absorbances at 535 and 572 nm on a microplate spectrophotometer reader (Tecan; Männedorf, Switzerland) and the results are reported as nM/mg of protein [31].

### 2.14. Superoxide Anion Production

The quantitation of superoxide anion production in tissue homogenates (10 mg/mL in 1.15% KCl) was performed using the nitro blue tetrazolium (NBT) assay [32]. Briefly, 50 *μ*L of homogenate was incubated with 100 *μ*L of NBT (1 mg/mL; Sigma-Aldrich, St. Louis, MO, USA) in 96-well plates at 37°C for 1 hr. The supernatant was then carefully removed and the reduced formazan solubilized by adding 120 *μ*L of 2 M KOH and 140 *μ*L of DMSO. The NBT reduction was measured at 600 nm using a microplate spectrophotometer reader (Tecan; Männedorf, Switzerland). The protein content was used for data normalization.

### 2.15. Histopathology

Samples from dorsal back skins, spleen, and left submandibular LN were separated and fixed in 10% neutral buffered formalin and then embedded in paraffin, sectioned (3~4 *μ*m), and stained with hematoxylin and eosin (H&E) for general histopathology, Masson's trichrome (MT) for collagen fiber, or toluidine blue for mast cells according to our established methods [27], and after that the histopathological profiles of each sample were observed under light microscope (Nikkon, Tokyo, Japan). To detail changes more, mean epithelial thicknesses in the epidermis (*μ*m), mean numbers of inflammatory and mast cells infiltrated in the dermis (cells/mm^2^ of dermis), total splenic thicknesses (mm/central regions), mean numbers of white pulp (white pulps/mm^2^ of splenic parenchyma) and red pulp cells (×10^3^ cells/mm^2^ of splenic parenchyma), total submandibular LN thicknesses (mm/central regions), cortex lymphoid follicle numbers (follicles/mm^2^ of cortex), and mean thicknesses (*μ*m/LN) of submandibular LN were calculated for general histomorphometric analysis using a computer-assisted image analysis program (*i*Solution FL ver. 9.1, IMT *i*-solution Inc., Quebec, Canada) under H&E stain with collagen fiber occupied regions in the dermis (%/mm^2^ of dermis) under MT stain, according to our previously established methods [25, 27], respectively. The histopathologist was blinded to group distribution when this analysis was made.

### 2.16. Immunohistochemistry

After deparaffinization of prepared skin, submandibular LN, or spleen histological paraffin sections, citrate buffer antigen (epitope) retrieval pretreatment was conducted as previously [27]. Briefly, preheat water bath with staining dish containing 10 mM citrate buffers (pH 6.0) until temperature reaches 95–100°C. Immerse slides in the staining dish and place the lid loosely on the staining dish. Allow incubation for 20 minutes and turn off the water bath. Place the staining dish at room temperature and allow the slides to cool for 20 minutes. After epitope retrievals, sections were immunostained using avidin-biotin complex (ABC) methods for caspase-3, PARP, NT, 4-HNE, MMP-9, IFN-*γ*, iNOS, IL-1*β*, IL-2, and TNF-*α* according to the our previous studies [27, 33, 34]. Briefly, endogenous peroxidase activity was blocked by incubation in methanol and 0.3% H_2_O_2_ for 30 minutes, and nonspecific binding of immunoglobulin was blocked with normal horse serum blocking solution (Vector Lab., Burlingame, CA, USA, dilution 1 : 100) for 1 hr in humidity chamber. Primary antiserum was treated for overnight at 4°C in humidity chamber and then incubated with biotinylated universal secondary antibody (Vector Lab., Burlingame, CA, USA, dilution 1 : 50) and ABC reagents (Vectastain Elite ABC Kit, Vector Lab., Burlingame, CA, USA, dilution 1 : 50) for 1 hr at room temperature in humidity chamber. Finally, they were incubated in peroxidase substrate reagents (Vector Lab., Burlingame, CA, USA) for 3 min at room temperature. All sections were rinsed in 0.01 M PBS for 3 times, between steps. The cells or fibers occupied by over 30% of immunoreactivities, the density, of each antiserum, for caspase-3, PARP, NT, 4-HNE, MMP-9, IFN-*γ*, iNOS, IL-1*β*, IL-2, and TNF-*α* as compared with intact dermal keratinocytes or dermal fibers, were regarded as positive, and the mean numbers of caspase-3, PARP, NT, and 4-HNE immunoreactive cells in the epidermis (cells/100 epithelial cells), and mean IFN-*γ*, iNOS, IL-1*β*, IL-2, and TNF-*α* immunolabeled cell numbers in the dermis (cells/mm^2^ of dermis), spleen (cells/mm^2^ of spleen), and submandibular LN (cells/mm^2^ of LN) were also counted using an automated image analysis process as our established methods [27, 33, 34] with some of our modifications, respectively. In addition, the occupied percentages by MMP-9 immunoreactive fibers were also calculated in the dermis (%/mm^2^ of dermis), as MMP-9 immunoreactivities, in this experiment. The histopathologist was blinded to the group distribution when performing the analysis.

### 2.17. Statistical Analyses

All data were expressed as mean ± standard deviation (SD) of eight hairless mice. Multiple comparison tests for different dose groups were conducted. Variance homogeneity was examined using the Levene test. If the Levene test indicated no significant deviations from variance homogeneity, the obtained data were analyzed by one-way ANOVA test followed by least-significant differences multicomparison (LSD) test to determine which pairs of group comparison were significantly different. In case of significant deviations from variance homogeneity was observed at Levene test, a nonparametric comparison test; Kruskal-Wallis *H* test was conducted. When a significant difference is observed in the Kruskal-Wallis *H* test, the Mann-Whitney *U* (MW) test was conducted to determine the specific pairs of group comparison, which are significantly different. Statistical analyses were conducted using SPSS for Windows (Release 14.0K, IBM SPSS Inc., Armonk, NY, USA). In addition, the percent changes between intact vehicle and DNCB control were calculated to observe the severities of AD-like lesions induced by DNCB in this study, and the percent changes as compared with DNCB control and hairless mice bathing in seawaters or 1% DEXA topically applied mice were also calculated to help in the understanding of the efficacy, as follows according to our previous report [35], respectively:

(4) Percentage changes as compared with intact vehicle control%=Data of DNCB control−Data of intact vehicle control miceData of intact vehicle control mice×100,

(5) Percentage changes as compared with DNCB control %=Data of test material treated mice−Data of DNCB control miceData of DNCB contol mice×100.

## 3. Result

### 3.1. Changes on the Body Weight and Gains

**Figure 2:**
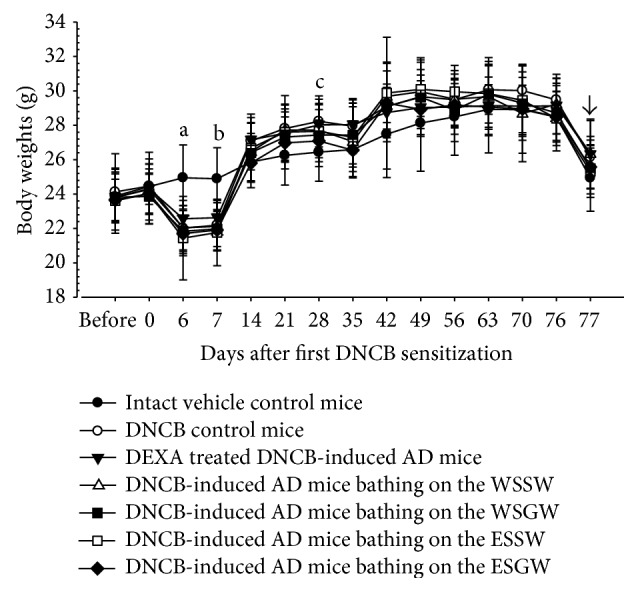
Body weights changes during 6 weeks of continuous bathing on seawaters or topical application of DEXA in DNCB-induced AD mice. Significant (*p* < 0.01) decreases of body weights were demonstrated in DNCB control mice at 6 and 7 days after initial DNCB sensitization as compared with intact vehicle control mice (a, b), but significantly (*p* < 0.05) increased body weights were transiently noticed at 3 weeks after first DNCB boosting in DNCB control mice as compared with intact vehicle control mice (c). In addition, topical application of 1% DEXA and bathing on the all four different seawaters also did not influence the body weights as compared with those of DNCB control mice, throughout all experimental periods. Values are expressed as mean ± SD of eight hairless mice. AD = allergic/atopic-like dermatitis; DNCB = 2,4-dinitrochlorobenzene; DEXA = dexamethasone-water soluble; WSSW = west surface seawater collected around Wepo-ri (Ganghwa-do, Republic of Korea); WSGW = west saline groundwater collected at Yonggungoncheon (Seokmo-do, Republic of Korea); ESSW = east surface seawater collected around Nagok-ri (Uljin, Republic of Korea); ESGW = east saline groundwater collected around Hoojeong-ri (Uljin, Republic of Korea). Before mean 1 day before initial DNCB sensitization application; the day of 7 means start day of DNCB sensitization; the day of 35 means start day of bathing or topical treatment of DEXA. All animals were overnight fasted before sacrifice (arrow).

Significantly lower body weights were demonstrated in DNCB control mice at 6 and 7 days after initial DNCB sensitization than in intact vehicle control mice (*p* < 0.01), but significantly higher body weights were transiently noticed at 3 weeks after the first DNCB boosting in DNCB control mice than in intact vehicle control mice (*p* < 0.05). Therefore, there were no significant changes in body weight gain during the total 11-week experimental period or during the 6-week bathing periods between DNCB control mice and intact vehicle control mice. In addition, neither topical application of 1% dexamethasone (DEXA) nor bathing with all four seawaters influenced the body weight or body weight gain compared to the DNCB control mice throughout the entire experimental period (Figure 2).

### 3.2. Clinical Skin Severity Score Changes

Among all of the animals, AD-induced mice were selected 1 day before the initial treatment of DEXA or bathing according to the 10 clinical skin severity scores in the categories of pruritus/itching, erythema/hemorrhage, edema, excoriation/erosion, and scaling/dryness. The clinical skin severity scores were significantly higher in the DNCB control mice than in the intact vehicle control mice from 24 h before the initial bathing or topical application of 1% DEXA to the end of the experimental period. However, these increases in clinical skin severity scores were significantly lower in the intact vehicle control mice than in the DNCB control mice from 1 week after the initial topical application of 1% DEXA or bathing in WSGW (*p* < 0.01 and *p* < 0.05, resp.). In addition, significantly lower clinical skin severity scores were detected in the intact vehicle control mice than in the DNCB control mice from 2 weeks after initial bathing with ESGW and from 3 weeks after initial bathing with WSSW and ESSW (*p* < 0.01 and *p* < 0.05, resp.) (Table 2).

### 3.3. Changes on the Scratching Behaviors

As noted above, AD-induced mice were selected 1 day before the initial treatment of DEXA or bathing; 4 weeks after the initial DNCB boosting, those with >300 episodes of head-scratching behavior per 30 min along with increases in the 10 clinical skin severity scores were selected. DNCB control mice showed significantly greater increases in scratching behavior than intact vehicle control mice from 24 h before initial bathing or topical application of 1% DEXA until the end of the experimental period (*p* < 0.01). However, these increases in scratching behavior were significantly less marked in intact vehicle control mice than in DNCB control mice from 1 week after the initial topical application of 1% DEXA until the end of the experimental period (*p* < 0.01 or *p* < 0.05). In addition, significantly less scratching behavior was detected in intact vehicle control mice than in DNCB control mice from 2 weeks after the initial bathing in WSGW and ESGW and from 4 weeks after the initial bathing in WSSW and ESSW until the end of the experimental period (*p* < 0.01 and *p* < 0.05, resp.) (Table 3).

### 3.4. Effects on the Serum Total IgE Levels

**Figure 3:**
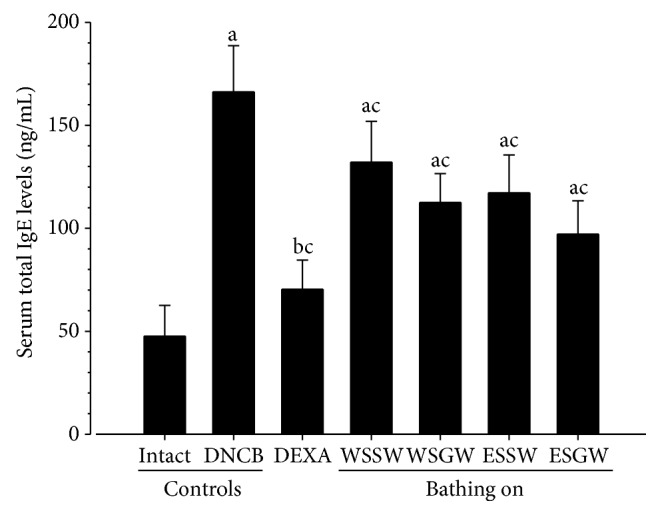
Changes of the serum IgE levels after 6 weeks of continuous bathing on seawaters or topical application of DEXA in DNCB-induced AD mice. Significant (*p* < 0.01) increases of serum total IgE levels were detected in DNCB control mice as compared with intact vehicle control hairless mice. However, significant (*p* < 0.01) decreases of serum total IgE levels were detected by topical treatment of 1% DEXA, bathing on the ESGW, WSGW, ESSW, and WSSW as compared with DNCB control mice, in that order, respectively. Values are expressed as mean ± SD of eight hairless mice. AD = allergic/atopic-like dermatitis; DNCB = 2,4-dinitrochlorobenzene; DEXA = dexamethasone-water soluble; WSSW = west surface seawater collected around Wepo-ri (Ganghwa-do, Republic of Korea); WSGW = west saline groundwater collected at Yonggungoncheon (Seokmo-do, Republic of Korea); ESSW = east surface seawater collected around Nagok-ri (Uljin, Republic of Korea); ESGW = east saline groundwater collected around Hoojeong-ri (Uljin, Republic of Korea); Ig = immunoglobulin. ^a^ *p* < 0.01 and ^b^ *p* < 0.05 as compared with intact control by LSD test; ^c^ *p* < 0.01 as compared with DNCB control by LSD test.

Significantly higher serum total IgE levels were detected in DNCB control mice than in intact vehicle control hairless mice (*p* < 0.01). However, significantly lower serum total IgE levels were detected in intact vehicle control mice than in DNCB control mice after topical treatment with 1% DEXA and bathing in ESGW, WSGW, ESSW, and WSSW, in that order (*p* < 0.01) (Figure 3). The serum total IgE levels in DNCB control mice were 249.23% higher than those in the intact vehicle control mice, and these levels decreased by 57.63%, 20.50%, 32.30%, 29.46%, and 41.55% after topical application of 1% DEXA and bathing in WSSW, WSGW, ESSW, and ESGW, respectively.

### 3.5. Changes on the Submandibular LN and Spleen Weights

Significantly higher submandibular lymph node (LN) and absolute and relative spleen weights were detected in DNCB control mice than in intact control hairless mice (*p* < 0.01). However, significantly lower absolute and relative submandibular LN and spleen weights were detected in intact control hairless mice than in DNCB control mice after topical treatment with 1% DEXA and bathing in ESGW, WSGW, ESSW, and WSSW, in that order (*p* < 0.01) (Table 4).

### 3.6. Effects on the Splenic Cytokine Contents

Significantly higher splenic tissue levels of TNF-*α*, IL-1*β*, and IL-10 were detected in DNCB control mice than in intact control hairless mice (*p* < 0.01). However, significantly lower splenic tissue levels of TNF-*α*, IL-1*β*, and IL-10 were detected in intact control hairless mice than in DNCB control mice after topical treatment with 1% DEXA and bathing in ESGW, WSGW, ESSW, and WSSW, in that order (*p* < 0.01 and *p* < 0.05, resp.) (Table 5).

### 3.7. Changes on the Skin Tissue Cytokine mRNA Expressions (RT-PCR Analysis)

Significantly higher TNF-*α*, IL-4, IL-5, and IL-13 mRNA expression levels in the dorsal back skin were detected in DNCB control mice compared to intact control hairless mice, as determined by RT-PCR analysis (*p* < 0.01). However, significantly lower TNF-*α*, IL-4, IL-5, and IL-13 mRNA expression levels in the dorsal back skin were detected in intact control hairless mice than in DNCB control mice after topical treatment with 1% DEXA and bathing in ESGW, WSGW, ESSW, and WSSW, in that order (*p* < 0.01 and *p* < 0.05, resp.) (Table 6).

### 3.8. Effects on the Skin Tissue Antioxidant Defense Systems

Significantly lower glutathione (GSH) levels and significantly higher lipid peroxidation and superoxide anion production levels in the dorsal back skin were detected in DNCB control mice than in intact control hairless mice (*p* < 0.01). However, significantly higher GSH levels and lower lipid peroxidation and superoxide anion production levels in the dorsal back skin were detected in intact control hairless mice than in DNCB control mice after bathing in ESGW, WSGW, ESSW, and WSSW, in that order (*p* < 0.01 or *p* < 0.05). Topical application of 1% DEXA did not influence the skin tissue antioxidant defense system in intact control hairless mice compared to DNCB control mice (Table 7).

### 3.9. Histopathological Changes on the Dorsal Back Skin Tissues

**Figure 4:**
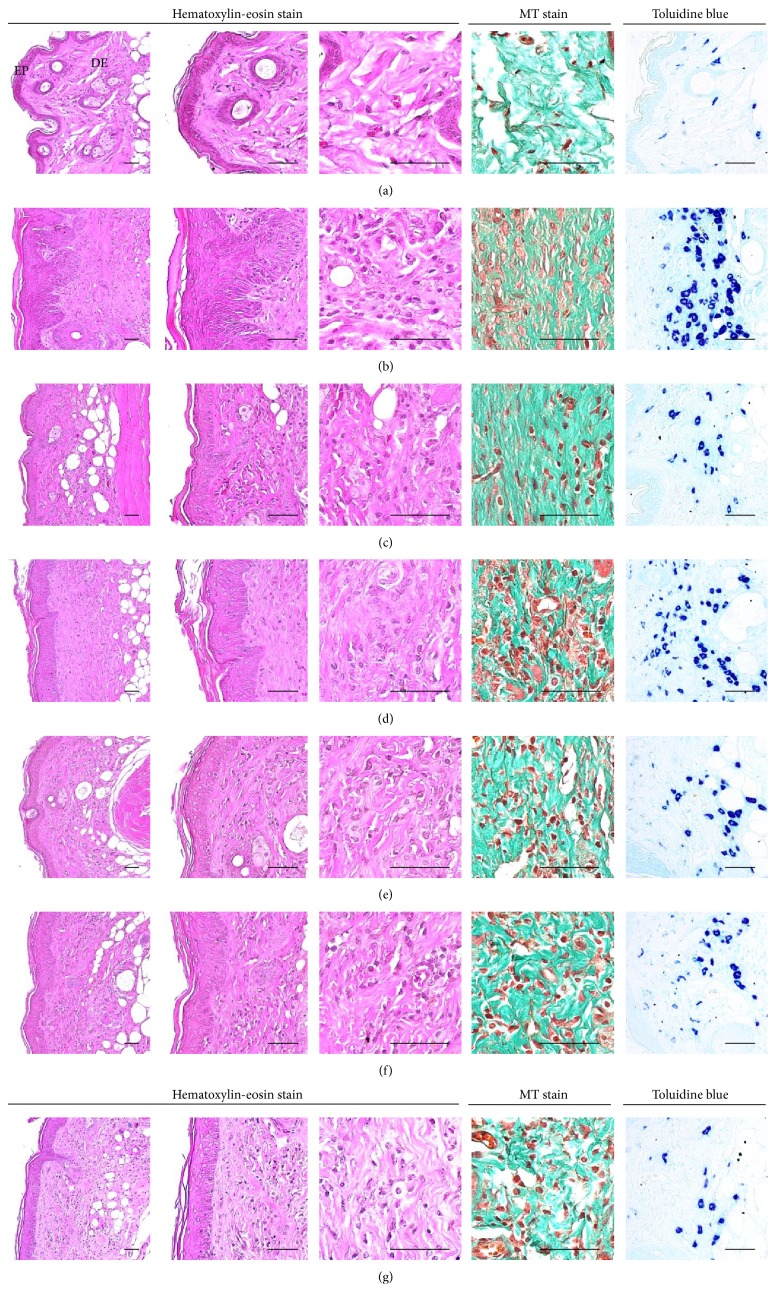
Representative histological images of dorsal back skin tissues, taken from unexposed intact or DNCB-induced AD mice bathing on seawaters or topical application of DEXA. Marked increases of mean epithelial thicknesses due to hyperplasia/hypertrophy of epidermal keratinocytes were detected on the dorsal back skin tissues in DNCB control mice with noticeable increases of the numbers of mast and inflammatory cells infiltrated into dermis and abnormal collagen depositions, respectively. However, these histopathological hypersensitivities related AD signs were significantly inhibited by bathing on the ESGW, WSGW, ESSW, and WSSW as compared with DNCB control mice, in that order, respectively. Topical application of 1% DEXA also significantly reduced the increases of mean epithelial thicknesses, numbers of dermal infiltrated mast, and inflammatory cells induced by NDCB treatment but did not influence the percentages of collagen fiber occupied dermal regions as compared with DNCB control mice, in this experiment. (a) Intact vehicle control mice bathing on the distilled water; (b) DNCB control mice bathing on the distilled water; (c) AD mice bathing on the WSSW; (d) AD mice bathing on the WSGW; (e) AD mice bathing on the ESSW; (f) AD mice bathing on the ESGW; (g) 1% DEXA topically applied AD mice. AD = allergic/atopic-like dermatitis; DNCB = 2,4-dinitrochlorobenzene; DEXA = dexamethasone-water soluble; WSSW = west surface seawater collected around Wepo-ri (Ganghwa-do, Republic of Korea); WSGW = west saline groundwater collected at Yonggungoncheon (Seokmo-do, Republic of Korea); ESSW = east surface seawater collected around Nagok-ri (Uljin, Republic of Korea); ESGW = east saline groundwater collected around Hoojeong-ri (Uljin, Republic of Korea); EP = epidermis; DE = dermis; MT = Masson's trichrome. Scale bars = 40 *μ*m.

**Figure 5 fig5:**
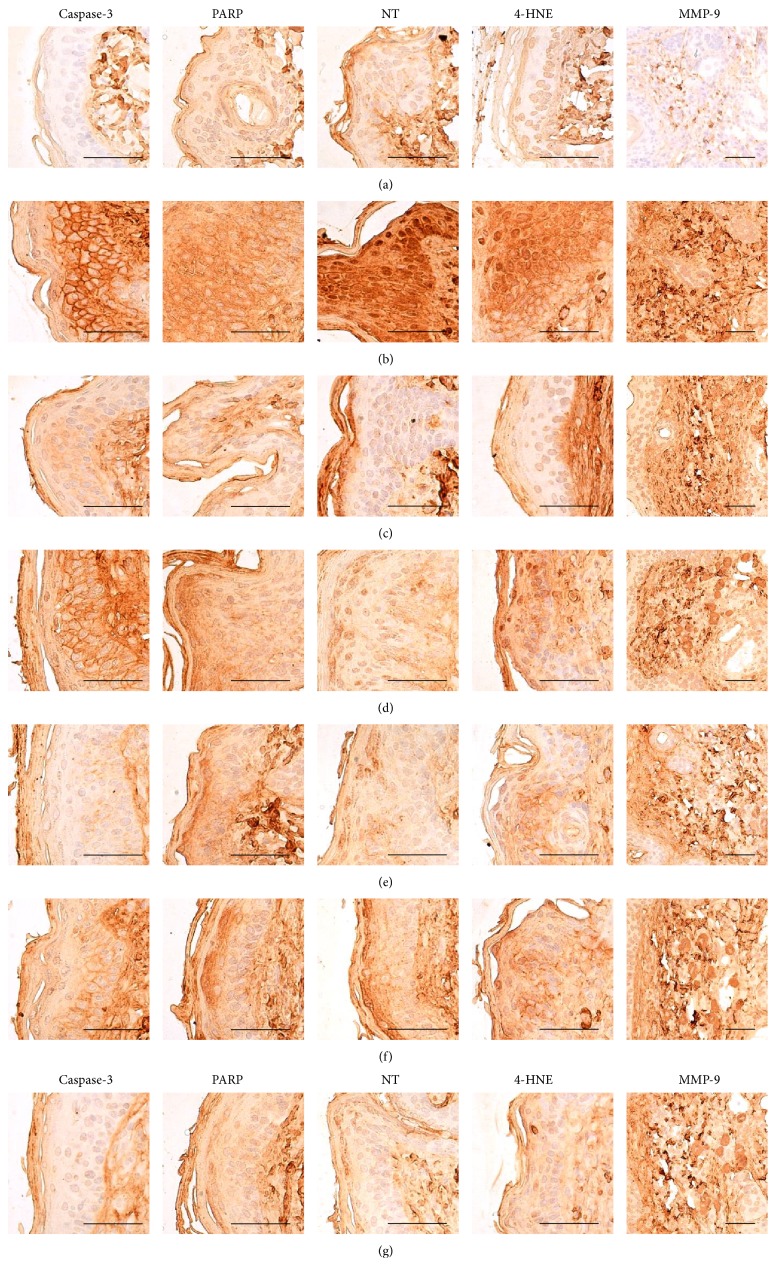
Representative immunohistochemical images of caspase-3, PARP, NT, 4-HNE, and MMP-9 in the dorsal back skin tissues, taken from unexposed intact or DNCB-induced AD mice bathing on seawaters or topical application of DEXA. Noticeable elevations of caspase-3, PARP, NT, and 4-HNE immunoreactive epidermal cells, dermal MMP-9 immunoreactivities were detected on the dorsal back skin tissues in DNCB control mice, respectively. However, these increases of immunoreactivities related AD signs were significantly inhibited by bathing on the ESGW, WSGW, ESSW, and WSSW as compared with DNCB control mice, in that order, respectively. Topical application of 1% DEXA also significantly reduced the increases of caspase-3, PARP, NT, and 4-HNE immunoreactive epidermal cells and dermal MMP-9 immunoreactivities as compared with DNCB control mice, in this experiment. (a) Intact vehicle control mice bathing on the distilled water; (b) DNCB control mice bathing on the distilled water; (c) AD mice bathing on the WSSW; (d) AD mice bathing on the WSGW; (e) AD mice bathing on the ESSW; (f) AD mice bathing on the ESGW; (g) 1% DEXA topically applied AD mice. AD = allergic/atopic-like dermatitis; DNCB = 2,4-dinitrochlorobenzene; DEXA = dexamethasone-water soluble; WSSW = west surface seawater collected around Wepo-ri (Ganghwa-do, Republic of Korea); WSGW = west saline groundwater collected at Yonggungoncheon (Seokmo-do, Republic of Korea); ESSW = east surface seawater collected around Nagok-ri (Uljin, Republic of Korea); ESGW = east saline groundwater collected around Hoojeong-ri (Uljin, Republic of Korea); PARP = cleaved poly (ADP-ribose) polymerase; NT = nitrotyrosine; 4-HNE = 4-hydroxynonenal; MMP = matrix metalloprotease; ABC = avidin-biotin complex. All being ABC immunostain. Scale bars = 40 *μ*m.

**Figure 6:**
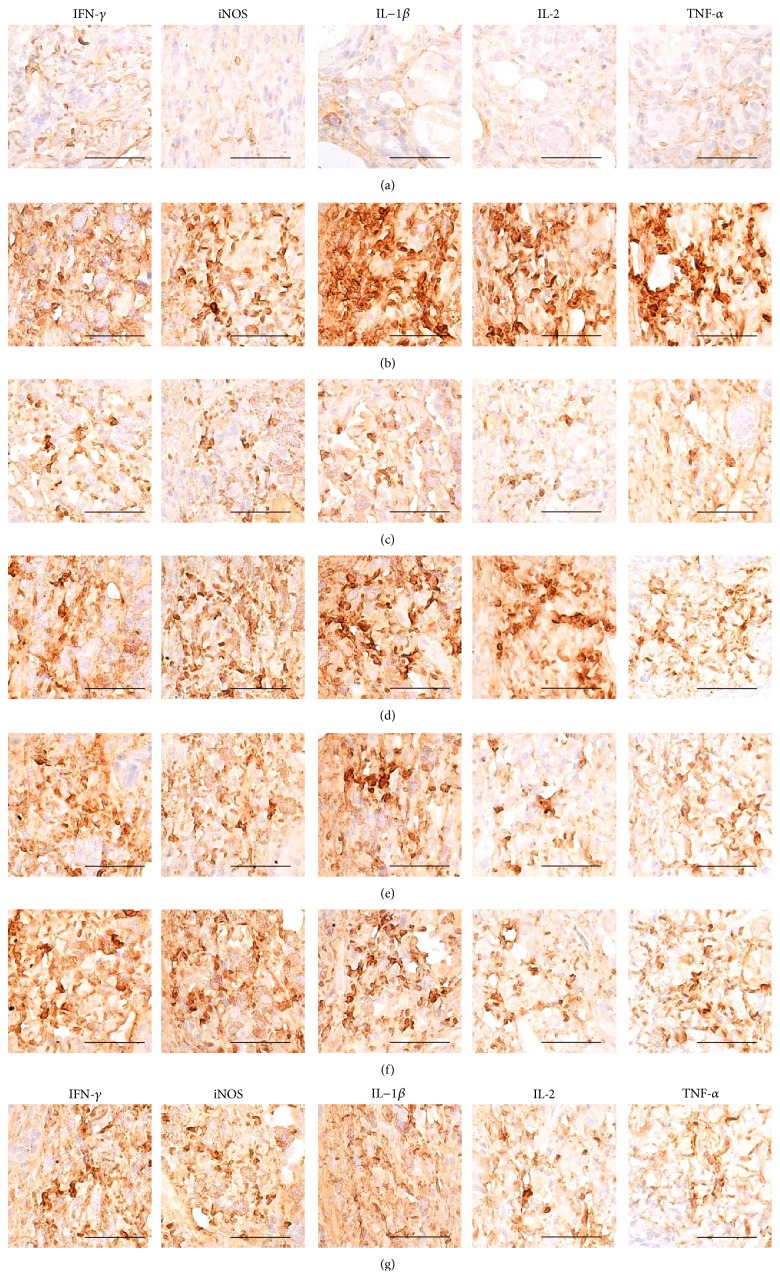
Representative immunohistochemical images of dermal IFN-*γ*, iNOS, IL-1*β*, IL-2, and TNF-*α* in the dorsal back skin tissues, taken from unexposed intact or DNCB-induced AD mice bathing on seawaters or topical application of DEXA. Dramatic infiltrations of dermal IFN-*γ*, iNOS, IL-1*β*, IL-2, and TNF-*α* immunolabeled cells were observed in DNCB control mice as compared with intact vehicle control hairless mice, respectively. However, these increases of immunoreactivities of IFN-*γ*, iNOS, IL-1*β*, IL-2, and TNF-*α* in the dermis were significantly inhibited by bathing on the ESGW, WSGW, ESSW, and WSSW as compared with DNCB control mice, in that order, respectively. Topical application of 1% DEXA also significantly reduced the increases of dermal IFN-*γ*, iNOS, IL-1*β*, IL-2, and TNF-*α* immunolabeled cells induced by NDCB treatment as compared with DNCB control mice, in this experiment. (a) Intact vehicle control mice bathing on the distilled water; (b) DNCB control mice bathing on the distilled water; (c) AD mice bathing on the WSSW; (d) AD mice bathing on the WSGW; (e) AD mice bathing on the ESSW; (f) AD mice bathing on the ESGW; (g) 1% DEXA topically applied AD mice. AD = allergic/atopic-like dermatitis; DNCB = 2,4-dinitrochlorobenzene; DEXA = dexamethasone-water soluble; WSSW = west surface seawater collected around Wepo-ri (Ganghwa-do, Republic of Korea); WSGW = west saline groundwater collected at Yonggungoncheon (Seokmo-do, Republic of Korea); ESSW = east surface seawater collected around Nagok-ri (Uljin, Republic of Korea); ESGW = east saline groundwater collected around Hoojeong-ri (Uljin, Republic of Korea); IFN = interferon; IL = interleukin; iNOS = inducible nitric oxide synthase (2); TNF = tumor necrosis factor; ABC = avidin-biotin complex. All being ABC immunostain. Scale bars = 40 *μ*m.

Histopathological signs of AD-related hypersensitivity were significantly lower in intact control hairless mice than in DNCB control mice after bathing in ESGW, WSGW, ESSW, and WSSW, in that order (*p* < 0.01 or *p* < 0.05). Topical application of 1% DEXA also resulted in significantly lower increases in the mean epithelial thicknesses, numbers of mast and inflammatory cells infiltrating the dermis, caspase-3, PARP, NT, and 4-HNE immunoreactive epidermal cells, dermal MMP-9 immunoreactivity, dermal IFN-*γ*, iNOS, IL-1*β*, IL-2, and TNF-*α* immunolabeled cells (*p* < 0.01) but did not influence the percentages of collagen fiber occupied dermal regions (Tables 8 and 9, Figures 4 [Fig fig5]–6).

### 3.10. Histopathological Changes on the Splenic Tissues

**Figure 7:**
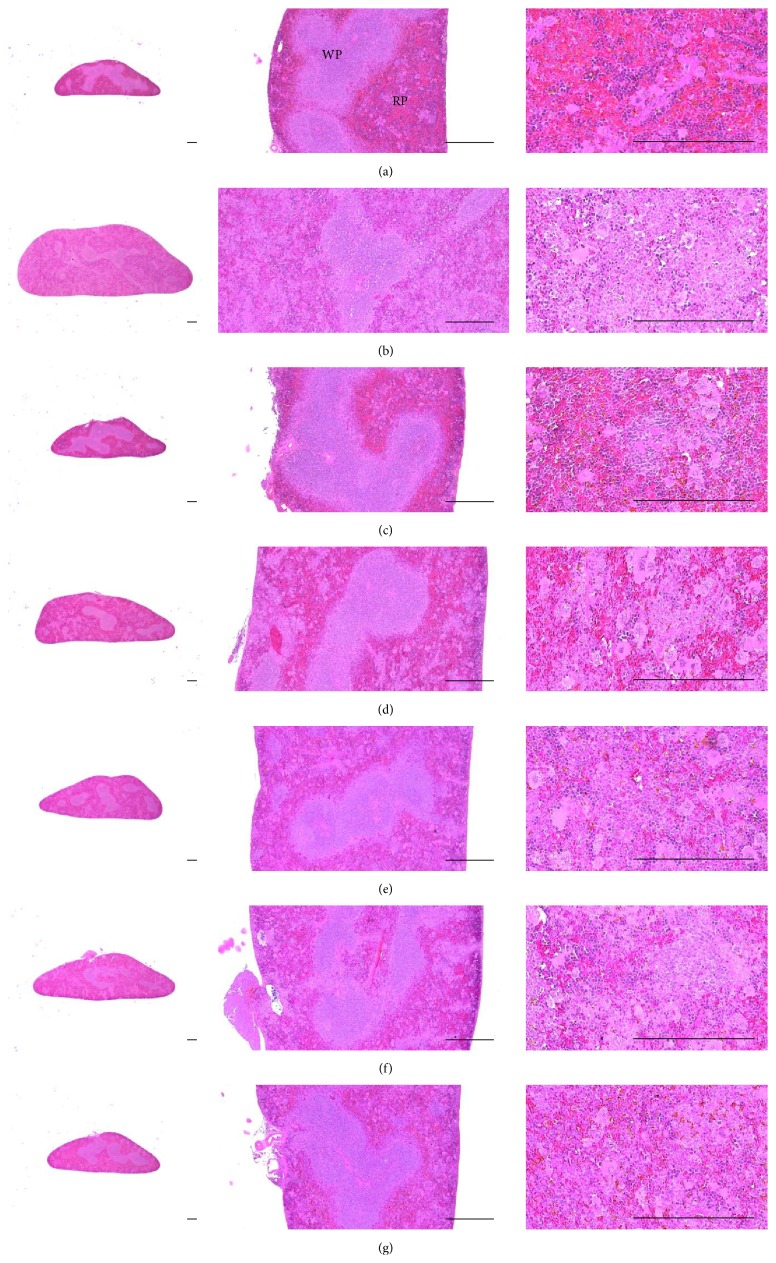
Representative histological images of splenic tissues, taken from unexposed intact or DNCB-induced AD mice bathing on seawaters or topical application of DEXA. Marked hypertrophic changes due to hyperplasia of red pulp lymphoid cells were detected on the splenic tissues in DNCB control mice as compared with intact vehicle control hairless mice, respectively. However, these hypersensitivities related splenic hypertrophic signs were significantly inhibited by topical treatment of 1% DEXA and bathing on the ESGW, WSGW, ESSW, and WSSW as compared with DNCB control mice, in that order, respectively. No meaningful changes on the numbers of white pulps were demonstrated by treatment of DNCB as compared with intact vehicle control and also by topical application of 1% DEXA or bathing on the all four types of seawaters as compared with DNCB control mice, in this experiment. (a) Intact vehicle control mice bathing on the distilled water; (b) DNCB control mice bathing on the distilled water; (c) AD mice bathing on the WSSW; (d) AD mice bathing on the WSGW; (e) AD mice bathing on the ESSW; (f) AD mice bathing on the ESGW; (g) 1% DEXA topically applied AD mice. AD = allergic/atopic-like dermatitis; DNCB = 2,4-dinitrochlorobenzene; DEXA = dexamethasone-water soluble; WSSW = west surface seawater collected around Wepo-ri (Ganghwa-do, Republic of Korea); WSGW = west saline groundwater collected at Yonggungoncheon (Seokmo-do, Republic of Korea); ESSW = east surface seawater collected around Nagok-ri (Uljin, Republic of Korea); ESGW = east saline groundwater collected around Hoojeong-ri (Uljin, Republic of Korea); WP = white pulp; RP = red pulp. All being hematoxylin-eosin stain. Scale bars = 400 *μ*m.

**Figure 8:**
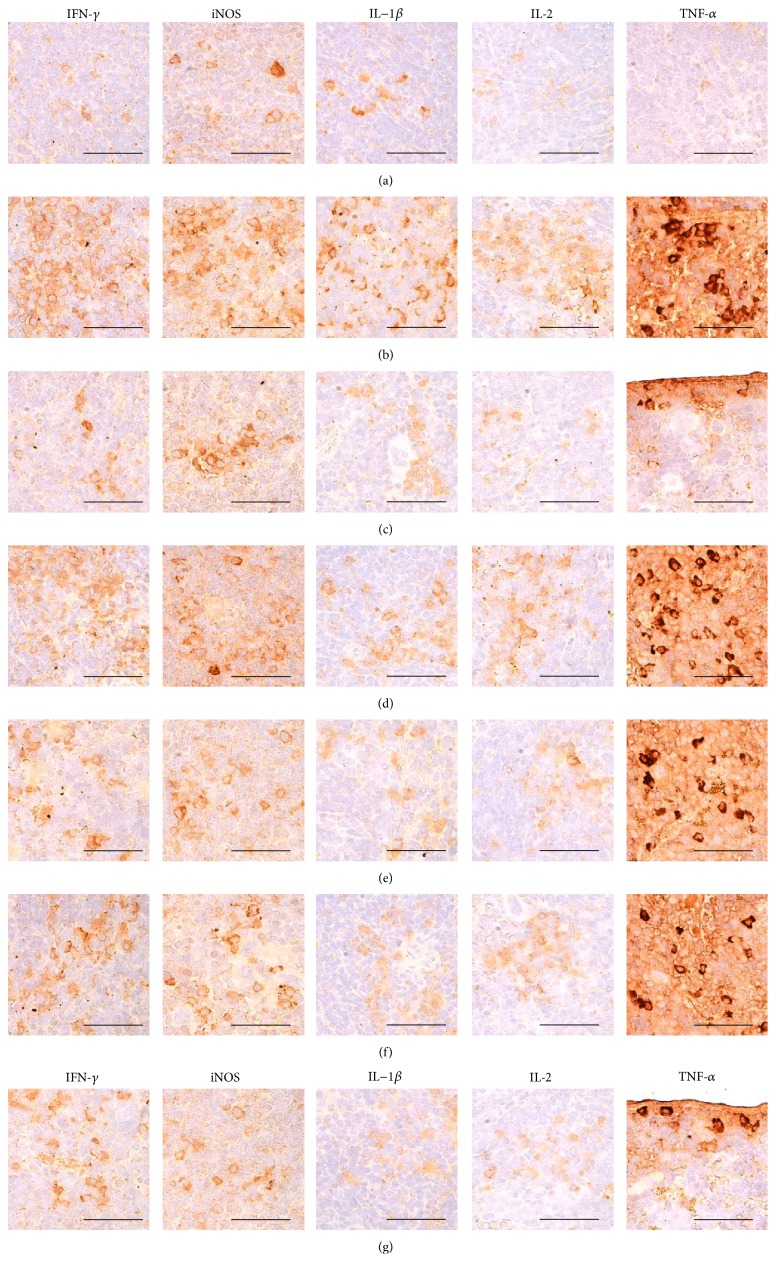
Representative immunohistochemical images of IFN-*γ*, iNOS, IL-1*β*, IL-2, and TNF-*α* in the splenic tissues, taken from unexposed intact or DNCB-induced AD mice bathing on seawaters or topical application of DEXA. Dramatic and noticeable increases of the numbers of IFN-*γ*, iNOS, IL-1*β*, IL-2, and TNF-*α* immunolabeled cells were observed in DNCB control mice as compared with intact vehicle control hairless mice, respectively. However, these hypersensitivities related increases of cytokine immunoreactive cells were significantly inhibited by topical treatment of 1% DEXA and bathing on the ESGW, WSGW, ESSW, and WSSW as compared with DNCB control mice, in that order, respectively. (a) Intact vehicle control mice bathing on the distilled water; (b) DNCB control mice bathing on the distilled water; (c) AD mice bathing on the WSSW; (d) AD mice bathing on the WSGW; (e) AD mice bathing on the ESSW; (f) AD mice bathing on the ESGW; (g) 1% DEXA topically applied AD mice. AD = allergic/atopic-like dermatitis; DNCB = 2,4-dinitrochlorobenzene; DEXA = dexamethasone-water soluble; WSSW = west surface seawater collected around Wepo-ri (Ganghwa-do, Republic of Korea); WSGW = west saline groundwater collected at Yonggungoncheon (Seokmo-do, Republic of Korea); ESSW = east surface seawater collected around Nagok-ri (Uljin, Republic of Korea); ESGW = east saline groundwater collected around Hoojeong-ri (Uljin, Republic of Korea); IFN = interferon; IL = interleukin; iNOS = inducible nitric oxide synthase (2); TNF = tumor necrosis factor; ABC = avidin-biotin complex. All being ABC immunostain. Scale bars = 40 *μ*m.

Significantly higher total splenic thickness, number of red pulp lymphoid cells, and numbers of IFN-*γ*, iNOS, IL-1*β*, IL-2, and TNF-*α* immunolabeled cells were observed in DNCB control mice than in intact vehicle control hairless mice (*p* < 0.01). However, these hypersensitivity-related signs of splenic hypertrophy were significantly lower in intact vehicle control hairless mice than in DNCB control mice after topical treatment of 1% DEXA and bathing in ESGW, WSGW, ESSW, and WSSW, in that order (*p* < 0.01 and *p* < 0.05, resp.). No meaningful changes in the number of white pulp cells were demonstrated after treatment with DNCB, topical application of 1% DEXA, or bathing in any of the four types of seawater (Table 10, Figures 7 and 8).

### 3.11. Histopathological Changes on the Submandibular LN Tissues

**Figure 9:**
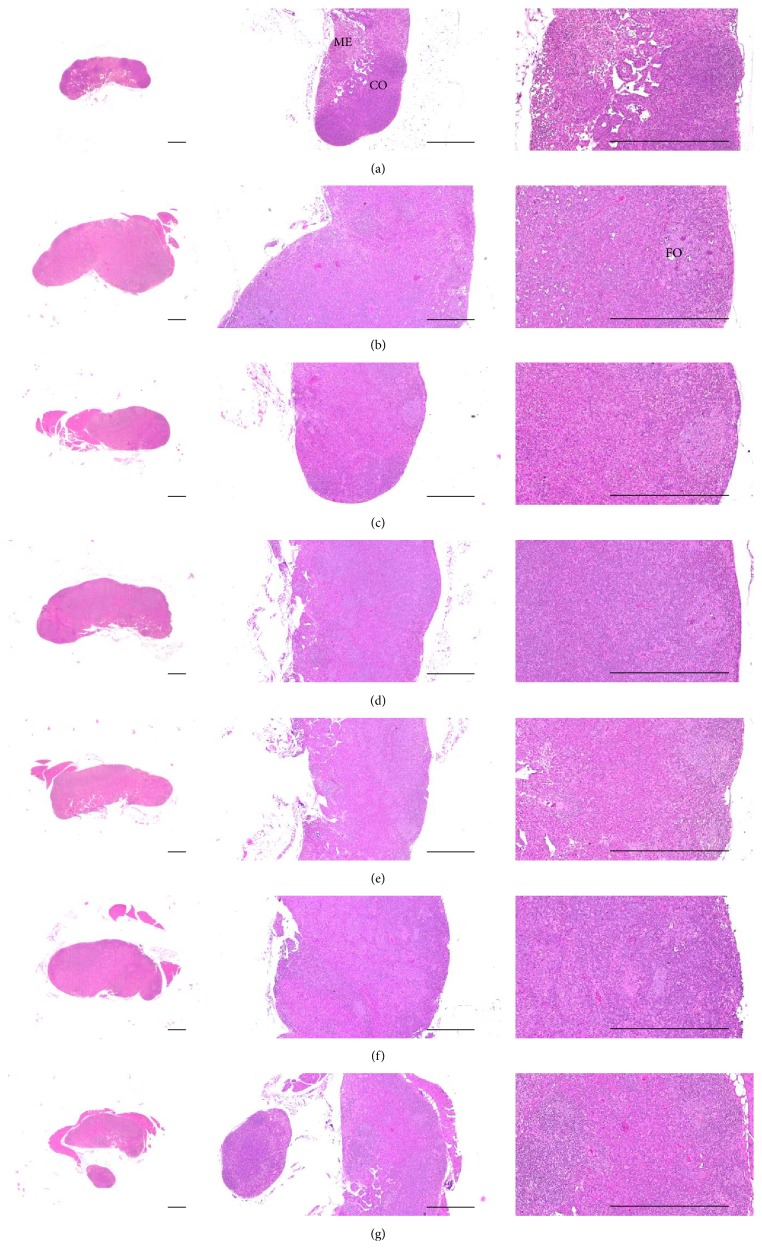
Representative histological images of submandibular LN tissues, taken from unexposed intact or DNCB-induced AD mice bathing on seawaters or topical application of DEXA. Noticeable hypertrophic changes due to hyperplasia of cortex lymphoid cells were detected on the submandibular LN tissues in DNCB control mice as compared with intact vehicle control hairless mice, respectively. However, these submandibular LN hypersensitivities related hypertrophic signs were significantly inhibited by topical treatment of 1% DEXA and bathing on the ESGW, WSGW, ESSW, and WSSW as compared with DNCB control mice, in that order, respectively. (a) Intact vehicle control mice bathing on the distilled water; (b) DNCB control mice bathing on the distilled water; (c) AD mice bathing on the WSSW; (d) AD mice bathing on the WSGW; (e) AD mice bathing on the ESSW; (f) AD mice bathing on the ESGW; (g) 1% DEXA topically applied AD mice. AD = allergic/atopic-like dermatitis; DNCB = 2,4-dinitrochlorobenzene; DEXA = dexamethasone-water soluble; WSSW = west surface seawater collected around Wepo-ri (Ganghwa-do, Republic of Korea); WSGW = west saline groundwater collected at Yonggungoncheon (Seokmo-do, Republic of Korea); ESSW = east surface seawater collected around Nagok-ri (Uljin, Republic of Korea); ESGW = east saline groundwater collected around Hoojeong-ri (Uljin, Republic of Korea); LN = lymph node; CO = cortex; FO = lymphoid follicle; ME = medulla. All being hematoxylin-eosin stain. Scale bars = 400 *μ*m.

**Figure 10:**
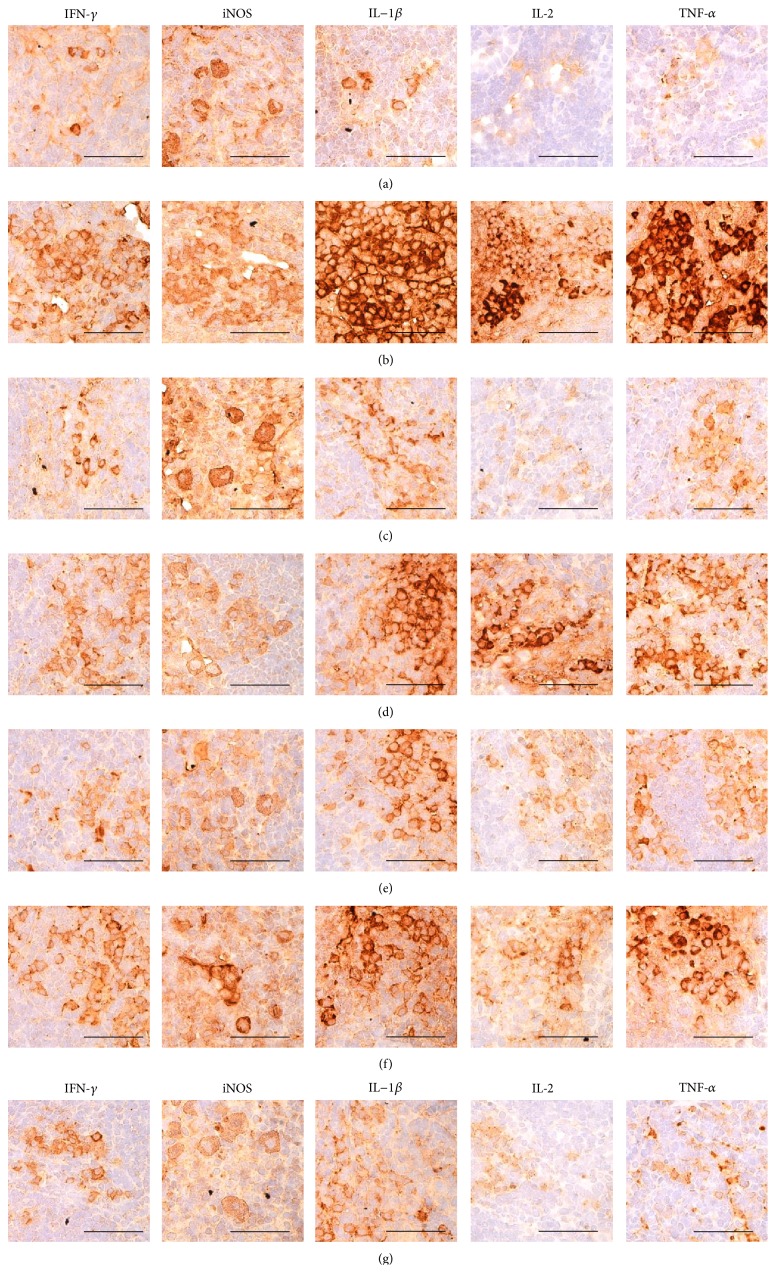
Representative immunohistochemical images of IFN-*γ*, iNOS, IL-1*β*, IL-2, and TNF-*α* in the submandibular LN tissues, taken from unexposed intact or DNCB-induced AD mice bathing on seawaters or topical application of DEXA. Noticeable increases of the numbers of IFN-*γ*, iNOS, IL-1*β*, IL-2, and TNF-*α* immunolabeled cells were observed in DNCB control mice as compared with intact vehicle control hairless mice, respectively. However, these submandibular LN hypersensitivities related increases of cytokine immunoreactive cells were significantly inhibited by topical treatment of 1% DEXA, bathing on the ESGW, WSGW, ESSW, and WSSW as compared with DNCB control mice, in that order, respectively. AD = allergic/atopic-like dermatitis; DNCB = 2,4-dinitrochlorobenzene; DEXA = dexamethasone-water soluble; WSSW = west surface seawater collected around Wepo-ri (Ganghwa-do, Republic of Korea); WSGW = west saline groundwater collected at Yonggungoncheon (Seokmo-do, Republic of Korea); ESSW = east surface seawater collected around Nagok-ri (Uljin, Republic of Korea); ESGW = east saline groundwater collected around Hoojeong-ri (Uljin, Republic of Korea); LN = lymph node; IFN = interferon; IL = interleukin; iNOS = inducible nitric oxide synthase (2); TNF = tumor necrosis factor; ABC = avidin-biotin complex. All being ABC immunostain. Scale bars = 40 *μ*m.

Significantly higher total submandibular LN thickness, number of cortex lymphoid follicles, cortex thickness, and numbers of IFN-*γ*, iNOS, IL-1*β*, IL-2, and TNF-*α* immunolabeled cells in the submandibular LN tissues were observed in DNCB control mice than in intact vehicle control hairless mice (*p* < 0.01). However, these hypersensitivity-related signs of submandibular LN hypertrophy were significantly inhibited by topical treatment with 1% DEXA and bathing in ESGW, WSGW, ESSW, and WSSW, in that order (*p* < 0.01) (Table 11, Figures 9 and 10).

## 4. Discussion

In this study, we examined the anti-AD effects of various types of seawater collected from different regions in Republic of Korea in a hairless mouse model of DNCB-induced AD after 6 weeks of bathing for 20 min once a day for 42 days. The results were compared to those after topical application of 1% DEXA.

Generally, marked decreases in body weight were noted during DNCB sensitization, but the body weights increased despite the development of hypersensitivity after chronic and repeated DNCB exposure. Therefore, DNCB sensitization and boosting did not critically influence the total body weight gains of mice [6, 36]. In the present study, significantly lower body weights were also demonstrated in DNCB control mice at 6 and 7 days after initial DNCB sensitization than in intact vehicle control mice, but significantly higher body weights were transiently noticed at 3 weeks after the first DNCB boosting in DNCB control mice than in intact vehicle control mice. Therefore, no significant changes in body weight gains were noted between the DNCB control mice and intact vehicle control mice during the total 11-week experimental period or 6-week bathing period; these findings are quite similar to those of previous studies [6, 36]. In addition, topical application of 1% DEXA and bathing in all four different seawaters did not influence the body weight or body weight gains compared to DNCB control mice throughout the entire experimental period.

The skin lesions in patients with AD are generally characterized by infiltration of various inflammatory cells such as mast cells, basophils, eosinophils, and T cells [1, 37]. AD is also associated with several common symptoms including itching, erythema, eczematous skin lesions, chronic relapse, and pruritus [2]. Application of DNCB onto the skin also causes inflammation and dermal sclerosis along with these common symptoms [38]. Therefore, clinical skin severity scores based on the five main skin lesions and symptoms (pruritus/itching, erythema/hemorrhage, edema, excoriation/erosion, and scaling/dryness) have been used as valuable predictor of progression of AD with scratching behavior [4]. Mast cells mediate inflammatory responses such as hypersensitivity and allergic reactions, and the allergen cross-linking of surface IgE-dependent mast cell activation stimulates degranulation and release of histamine, leukotrienes, proteases, prostaglandins, and cytokines [6]. Activated mast cells release a variety of inflammatory mediators following cross-linking of IgE-receptor complexes at the high-affinity IgE receptor I. Of these mediators, histamine is generally considered to be a marker of mast cell degranulation in immediate allergic reactions and is a potent inducer of itching. Histamine is a characteristic major mediator in mast cell storage granules and directly triggers type I allergic responses [6, 39]. In the present study, IgE-mediated hypersensitivity, dermal sclerosis, and inflammatory and mast cell infiltration induced by DNCB treatment were significantly decreased by bathing in ESGW, WSGW, ESSW, and WSSW, in that order, and by topical treatment of 1% DEXA. These findings suggest that bathing in these four different types of seawaters around Republic of Korea can inhibit the symptoms of DNCB-induced dermatitis.

The pathogenesis of AD is complex, involving genetic, environmental, and immunological factors. In particular, IL-4, IL-5, and IL-13, which are produced by Th2 cells, may have especially key roles in the onset and development of AD [40]. Although the etiology and pathology of AD are not fully understood, a recent study reported that typical symptoms of AD involve increased levels of Th2-mediated cytokines and a deficiency in Th1-mediated cytokines [41]. Th2 cells are dominant during the acute phase of AD, whereas Th1 cells are dominant and contribute to pathogenesis during the chronic phase [42]. An elevated IgE level is a hallmark of AD, and the expression of IL-4 contributes to this elevation. IL-4 stimulates IgE production in B cells. IgE released from B cells binds to mast cells, which then degranulate and release various biological mediators in patients with IgE-mediated AD [43]. AD is dependent upon the secretion of the cytokines IL-4, IL-5, and IL-13 by Th2 cells that are generated from precursors. Most patients with AD have increased eosinophils and IgE levels due to elevated IL-4, IL-5, and IL-13 produced by Th2 cells [44]. TNF-*α* is a well-known proinflammatory cytokine [45] and is markedly increased in DNCB-associated dermatitis [46]. IL-2 is normally produced by T cells during an immune response [47] and has a well-documented role in the induction of pruritus in AD [48]. IFN-*γ* is a 20 kDa to 25 kDa glycoprotein produced by CD8^+^ T cells and natural killer (NK) cells in response to IL-2. IFN-*γ* has complex effects on B- and T-cell functions and enhances NK cell and macrophage activities [49]. Increases in IFN-*γ* activities have also been observed in DNCB-induced dermatitis [50]. Increases in iNOS activities related to the proinflammatory agents endotoxin, IL-1*β*, TNF-*α*, and IFN-*γ* can induce shock and inflammatory responses in the body [51], and overexpression of iNOS is also involved in the pathogenesis of AD [52]. Therefore, downregulation of iNOS, IL-2, TNF-*α*, and IFN-*γ* expression has been used to predict the favorable effects of test materials in patients with various allergic diseases. In addition, AD induces systemic hypersensitivity and marked proliferation of central and peripheral lymphocytes, especially T cells [53]. In the present study, these systemic and local hypersensitivities induced by DNCB treatment were significantly inhibited by bathing in ESGW, WSGW, ESSW, and WSSW, in that order, as well as by topical treatment of 1% DEXA. These findings are considered to be direct evidence that bathing in these four different types of seawater in Republic of Korea inhibits the symptoms of DNCB-induced dermatitis through their potent systemic and local immunomodulatory effects. A previous study found that hydrotherapy can modulate lymphocyte and cytokine production [14].

AD leads to an imbalance between ROS and endogenous antioxidants, causing depletion of endogenous antioxidants such as GSH [18, 54]. GSH is a representative endogenous antioxidant that prevents tissue damage by maintaining low levels of ROS and acts as a protective antioxidant factor in tissues [55]. GSH also acts as a cofactor for glutathione peroxidase and glutathione reductase, which reduce hydrogen peroxide and lipid hydroperoxides [56]. 4-HNE is an *α*,*β*-unsaturated hydroxyalkenal produced by lipid peroxidation in cells and has been used as a valuable tissue lipid peroxidation marker. It is currently being considered a possible causal agent of numerous diseases [57]. NT is a product of tyrosine nitration mediated by reactive nitrogen species such as peroxynitrite anion and nitrogen dioxide. It is detected in many pathological conditions and is a marker of iNOS-dependent, reactive nitrogen species-induced nitrative stress [58, 59]. In the present study, depletion of endogenous antioxidants and increases in oxidative stress were significantly inhibited by bathing in ESGW, WSGW, ESSW, and WSSW, in that order. Hydrotherapy has potent antioxidant effects in various types of dermatitis [15–18]. In further support of the antioxidant effects of bathing in WSSW, WSGW, ESSW, and ESGW, all four types of seawater inhibited lipid peroxidation as determined by the MDA concentration of the TBARS assay [60]. Although epidermal NT and 4-HNE immunoreactive cells were also lower in mice treated with 1% DEXA than in DNCB control mice, 1% DEXA treatment did not influence the skin tissue GSH levels, lipid peroxidation, or superoxide anion production in this study.

Apoptosis occurs through two pathways: an extrinsic pathway involving the interaction of death ligands with their respective cell surface receptors and an intrinsic pathway initiated by insults that damage DNA, such as ultraviolet light and chemotherapeutic agents. Both pathways eventually result in mitochondrial damage with release of cytochrome *c* and downstream activation of caspases, such as caspase-3. Activation of other downstream caspases results in cleavage of cellular proteins, such as PARP, cytokeratin 18, and other caspases, leading to the morphologic and biochemical changes characteristic of apoptosis [61]. Therefore, caspase-3 with PARP has been used as an apoptotic marker. However, several studies have suggested that excessive activation of PARP results in necrosis due to the depletion of intracellular energy levels through the overconsumption of its substrate NAD. Regardless of the association of PARP with both apoptosis and necrosis, the inhibition of PARP is considered to be a useful strategy for prevention of various types of organ damage [62]. In addition, it has been reported that apoptosis is also involved in the skin damage seen in DNCB-induced AD [38]. In the present study, these increases in apoptotic markers, caspase-3, and PARP immunoreactivity induced by DNCB treatment were markedly inhibited by bathing in ESGW, WSGW, ESSW, and WSSW, in that order, as well as by topical treatment with 1% DEXA, and the changes corresponded with the epidermal histological changes. These findings suggest that bathing in the four different types of seawaters around Republic of Korea has potent keratinocyte protection activity against DNCB-induced epidermal apoptosis by modulating the activities of caspase-3 and PARP.

MMP expression is usually low in unstimulated skin cells or normal skin tissues, but the expression of some MMPs is induced by various extracellular stimuli, such as ultraviolet or infrared radiation, growth factors, cytokines, and tumor promoters [63, 64]. Recent studies have shown that several MMPs, especially MMP-8 and MMP-9, are significantly higher in patients with than without AD [65, 66]. Therefore, the available evidence suggests that MMPs may indeed play a part in the pathology of AD and that inhibition of MMP activity, either directly by a specific inhibitor or indirectly by reducing its expression, may be an effective therapeutic method of counteracting AD-related skin sclerosis [67]. In the present study, these increases in MMP-9 immunoreactivity and related abnormal dermal collagen deposition were markedly and significantly inhibited by bathing in ESGW, WSGW, ESSW, and WSSW, in that order. These findings suggest that bathing in these four different types of seawater in Republic of Korea has strong inhibitory activity against DNCB-induced skin sclerosis by modulating the activities of MMP-9.

## 5. Conclusion

The results obtained in this study suggest that bathing on the all four different types of seawaters collected around Republic of Korea has favorable protective effects against DNCB-induced AD through their favorable systemic and local immunomodulatory effects, active cytoprotective antiapoptotic effects, inhibitory effects of MMP activity, and anti-inflammatory and antioxidative effects, in the order of ESGW, WSGW, ESSW, and WSSW, at least in the condition of this experiment. These different efficacies along different collecting regions may be directly related to the different salinity and mineral compositions. Therefore, it is expected that bathing on the seawaters modulated their salinity and mineral compositions maybe serve as a predictable alternative therapy in AD patients in future.

## Figures and Tables

**Table 1:** Salinity and mineral compositions of individual seawaters used in this study.

Seawaters	Temperature (°C)	Depth (m)	Salinity (‰)	Mineral compositions (mg/L)			
				Ca	Mg	K	Na
GUGW	30		0.40	81.00	121.00	12.00	569.00
WSSW	22	5	26.20	234.61	923.67	481.34	7081.18
WSGW	70	700	22.40	3243.08	207.79	325.91	3850.67
ESSW	21.9	5	34.00	417.50	1264.00	383.00	10672.00
ESGW	21.7	689	26.00	1856.00	1012.00	38.00	3178.00

Seawaters were filtered with pore size 1.2 *μ*m GF/C Grass Microfiber Filter (Korea Filter Paper Co., Ltd., Seoul, Republic of Korea) except for WSGW, which was not filtered before being used in this experiment. GUGW = general underground water; WSSW = west surface seawater collected around Wepo-ri (Ganghwa-do, Republic of Korea); WSGW = west saline groundwater collected at Yonggungoncheon (Seokmo-do, Republic of Korea); ESSW = east surface seawater collected around Nagok-ri (Uljin, Republic of Korea); ESGW = east saline groundwater collected around Hoojeong-ri (Uljin, Republic of Korea).

**Table 2:** Changes on the clinical skin severity scores during 6 weeks of continuous bathing on seawaters or topical application of DEXA in DNCB-induced AD mice.

Index	Controls		DEXA	Bathing on			
	Intact	DNCB		WSSW	WSGW	ESSW	ESGW
Days after initial DNCB sensitization							
35	1.00 ± 0.76	12.13 ± 1.55^a^	12.25 ± 1.39^a^	12.25 ± 1.83^a^	12.00 ± 1.51^a^	12.13 ± 1.25^a^	12.25 ± 1.67^a^
38	1.13 ± 0.99	11.75 ± 1.28^a^	11.00 ± 1.51^a^	11.38 ± 1.41^a^	11.13 ± 0.99^a^	11.00 ± 1.20^a^	11.25 ± 1.67^a^
42	0.75 ± 0.71	11.50 ± 1.41^a^	8.75 ± 1.28^ab^	10.75 ± 1.04^a^	10.25 ± 1.16^ac^	10.50 ± 1.20^a^	10.38 ± 1.30^a^
49	1.00 ± 0.53	10.63 ± 1.41^a^	7.00 ± 1.20^ab^	9.88 ± 0.83^a^	8.88 ± 1.13^ab^	9.75 ± 1.16^a^	8.63 ± 1.06^ab^
56	1.00 ± 0.53	10.13 ± 1.46^a^	6.13 ± 1.13^ab^	9.00 ± 1.20^ac^	8.13 ± 0.99^ab^	9.00 ± 1.20^ac^	8.00 ± 1.07^ab^
63	1.25 ± 0.71	9.75 ± 1.49^a^	4.75 ± 1.49^ab^	7.75 ± 1.04^ab^	6.88 ± 1.25^ab^	7.63 ± 0.92^ab^	6.50 ± 0.93^ab^
70	1.13 ± 0.64	9.13 ± 1.46^a^	4.00 ± 1.31^ab^	7.25 ± 0.71^ab^	6.25 ± 1.04^ab^	6.88 ± 0.64^ab^	5.75 ± 0.71^a^
77	2.00 ± 0.76	8.38 ± 1.30^a^	3.50 ± 1.07^ab^	6.25 ± 0.71^ab^	5.25 ± 1.16^ab^	5.88 ± 0.64^ab^	4.88 ± 0.83^a^

Values are expressed as mean ± SD of eight hairless mice, scores (max = 15). AD = allergic/atopic-like dermatitis; DNCB = 2,4-dinitrochlorobenzene; DEXA = dexamethasone-water soluble; WSSW = west surface seawater collected around Wepo-ri (Ganghwa-do, Republic of Korea); WSGW = west saline groundwater collected at Yonggungoncheon (Seokmo-do, Republic of Korea); ESSW = east surface seawater collected around Nagok-ri (Uljin, Republic of Korea); ESGW = east saline groundwater collected around Hoojeong-ri (Uljin, Republic of Korea). ^a^ *p* < 0.01 as compared with intact control by LSD test; ^b^ *p* < 0.01 and ^c^ *p* < 0.05 as compared with DNCB control by LSD test.

**Table 3:** Changes on the scratching behaviors during 6 weeks of continuous bathing on seawaters or topical application of DEXA in DNCB-induced AD mice.

Index	Controls		DEXA	Bathing on			
	Intact	DNCB		WSSW	WSGW	ESSW	ESGW
Days after initial DNCB sensitization							
35	15.31 ± 9.09	478.13 ± 112.61^a^	480.88 ± 109.99^a^	484.50 ± 105.49^a^	480.63 ± 101.37^a^	475.50 ± 97.90^a^	481.88 ± 120.67^a^
38	13.63 ± 6.07	468.50 ± 111.31^a^	436.13 ± 101.11^a^	461.13 ± 106.48^a^	441.50 ± 87.29^a^	455.25 ± 93.97^a^	435.38 ± 88.52^a^
42	13.00 ± 4.41	456.25 ± 102.61^a^	324.88 ± 88.38^ac^	448.13 ± 101.73^a^	393.00 ± 52.97^a^	415.00 ± 76.14^a^	390.00 ± 77.26^a^
49	14.63 ± 6.67	448.63 ± 97.87^a^	223.25 ± 47.38^ab^	407.00 ± 85.47^a^	340.50 ± 42.52^ac^	369.50 ± 68.29^a^	432.00 ± 53.17^ac^
56	16.63 ± 5.42	431.25 ± 90.00^a^	177.50 ± 22.67^ab^	381.38 ± 83.58^a^	306.88 ± 27.97^ab^	355.63 ± 66.34^a^	299.38 ± 32.11^ab^
63	17.38 ± 5.60	412.38 ± 68.67^a^	149.25 ± 34.85^ab^	312.13 ± 66.85^ac^	290.38 ± 37.89^ab^	307.88 ± 39.20^ab^	273.63 ± 29.66^ab^
70	18.00 ± 4.28	410.50 ± 77.21^a^	90.63 ± 24.11^ab^	288.38 ± 70.04^ab^	251.75 ± 26.60^ab^	284.00 ± 39.37^ab^	229.75 ± 24.02^ab^
77	19.25 ± 5.06	405.00 ± 65.20^a^	70.88 ± 30.89^ab^	273.00 ± 63.19^ab^	230.25 ± 27.77^ab^	258.88 ± 30.68^ab^	203.75 ± 24.32^ab^

Values are expressed as mean ± SD of eight hairless mice, frequencies/30 min. AD = allergic/atopic-like dermatitis; DNCB = 2,4-dinitrochlorobenzene; DEXA = dexamethasone-water soluble; WSSW = west surface seawater collected around Wepo-ri (Ganghwa-do, Republic of Korea); WSGW = west saline groundwater collected at Yonggungoncheon (Seokmo-do, Republic of Korea); ESSW = east surface seawater collected around Nagok-ri (Uljin, Republic of Korea); ESGW = east saline groundwater collected around Hoojeong-ri (Uljin, Republic of Korea). ^a^ *p* < 0.01 as compared with intact control by MW test; ^b^ *p* < 0.01 and ^c^ *p* < 0.05 as compared with DNCB control by MW test.

**Table 4:** Changes on the lymphatic organ weights after 6 weeks of continuous bathing on seawaters or topical application of DEXA in DNCB-induced AD mice.

Groups	Absolute weights (g)		Relative weights (% of body weights)	
	Spleen	Submandibular LN	Spleen	Submandibular LN
Controls				
Intact	0.098 ± 0.014	0.007 ± 0.003	0.395 ± 0.069	0.029 ± 0.009
DNCB	0.237 ± 0.032^a^	0.019 ± 0.003^a^	0.909 ± 0.135^a^	0.073 ± 0.011^a^
Reference				
DEXA	0.123 ± 0.017^bc^	0.009 ± 0.002^c^	0.472 ± 0.082^c^	0.035 ± 0.00^bc^
Bathing on				
WSSW	0.192 ± 0.026^ac^	0.015 ± 0.003^ac^	0.730 ± 0.092^ac^	0.057 ± 0.007^ac^
WSGW	0.163 ± 0.027^ac^	0.013 ± 0.003^ac^	0.640 ± 0.094^ac^	0.052 ± 0.013^ac^
ESSW	0.177 ± 0.023^ac^	0.014 ± 0.003^ac^	0.705 ± 0.115^ac^	0.055 ± 0.015^ac^
ESGW	0.144 ± 0.021^ac^	0.012 ± 0.002^ac^	0.564 ± 0.076^ac^	0.046 ± 0.009^ac^

Values are expressed as mean ± SD of eight hairless mice, frequencies/30 min. AD = allergic/atopic-like dermatitis; DNCB = 2,4-dinitrochlorobenzene; DEXA = dexamethasone-water soluble; WSSW = west surface seawater collected around Wepo-ri (Ganghwa-do, Republic of Korea); WSGW = west saline groundwater collected at Yonggungoncheon (Seokmo-do, Republic of Korea); ESSW = east surface seawater collected around Nagok-ri (Uljin, Republic of Korea); ESGW = east saline groundwater collected around Hoojeong-ri (Uljin, Republic of Korea). ^a^ *p* < 0.01 and ^b^ *p* < 0.05 as compared with intact control by LSD test; ^c^ *p* < 0.01 as compared with DNCB control by LSD test.

**Table 5:** Changes on the splenic cytokine contents after 6 weeks of continuous bathing on seawaters or topical application of DEXA in DNCB-induced AD mice.

Groups	Splenic cytokine contents (pg/mg protein)		
	Tumor necrosis factor-*α*	Interleukin-1*β*	Interleukin-10
Controls			
Intact	164.92 ± 23.50	49.22 ± 12.57	216.95 ± 54.25
DNCB	530.62 ± 113.24^c^	196.31 ± 21.62^a^	688.86 ± 131.07^c^
Reference			
DEXA	198.70 ± 19.33^de^	87.83 ± 14.66^ab^	317.61 ± 52.05^ce^
Bathing on			
WSSW	373.00 ± 39.06^ce^	150.59 ± 18.60^ab^	514.88 ± 103.99^cf^
WSGW	322.99 ± 67.87^ce^	125.91 ± 14.36^ab^	443.48 ± 76.07^ce^
ESSW	352.92 ± 64.20^ce^	141.80 ± 21.29^ab^	508.11 ± 73.76^ce^
ESGW	296.31 ± 65.51^ce^	114.18 ± 23.27^ab^	402.62 ± 71.39^ce^

Values are expressed as mean ± SD of eight hairless mice. AD = allergic/atopic-like dermatitis; DNCB = 2,4-dinitrochlorobenzene; DEXA = dexamethasone-water soluble; WSSW = west surface seawater collected around Wepo-ri (Ganghwa-do, Republic of Korea); WSGW = west saline groundwater collected at Yonggungoncheon (Seokmo-do, Republic of Korea); ESSW = east surface seawater collected around Nagok-ri (Uljin, Republic of Korea); ESGW = east saline groundwater collected around Hoojeong-ri (Uljin, Republic of Korea); LSD = least-significant differences multi-comparison. ^a^ *p* < 0.01 as compared with intact control by LSD test; ^b^ *p* < 0.01 as compared with DNCB control by LSD test; ^c^ *p* < 0.01 and ^d^ *p* < 0.05 as compared with intact control by MW test; ^e^ *p* < 0.01 and ^f^ *p* < 0.05 as compared with DNCB control by MW test.

**Table 6:** Changes on the skin mRNA expressions after 6 weeks of continuous bathing on seawaters or topical application of DEXA in DNCB-induced AD mice.

Groups	Skin mRNA expressions (relative expressions/GAPDH mRNA)			
	Spleen	Submandibular LN	Spleen	Submandibular LN
Controls				
Intact	1.03 ± 0.08	1.01 ± 0.05	1.04 ± 0.11	1.01 ± 0.08
DNCB	5.62 ± 1.22^a^	4.94 ± 0.74^a^	5.50 ± 1.14^a^	3.07 ± 0.54^a^
Reference				
DEXA	2.74 ± 1.20^ab^	1.88 ± 0.56^ab^	2.25 ± 0.52^ab^	1.40 ± 0.21^ab^
Bathing on				
WSSW	4.18 ± 0.52^ac^	3.87 ± 0.60^ac^	3.95 ± 0.89^ab^	2.30 ± 0.37^ab^
WSGW	3.46 ± 0.77^ac^	3.06 ± 0.50^ab^	3.47 ± 0.82^ab^	1.98 ± 0.26^ab^
ESSW	3.94 ± 0.62^ac^	3.59 ± 0.66^ab^	3.70 ± 0.71^ab^	2.16 ± 0.28^ab^
ESGW	2.82 ± 1.22^ac^	2.55 ± 0.79^ab^	3.13 ± 0.63^ab^	1.74 ± 0.34^ab^

Values are expressed as mean ± SD of eight hairless mice. AD = allergic/atopic-like dermatitis; DNCB = 2,4-dinitrochlorobenzene; DEXA = dexamethasone-water soluble; WSSW = west surface seawater collected around Wepo-ri (Ganghwa-do, Republic of Korea); WSGW = west saline groundwater collected at Yonggungoncheon (Seokmo-do, Republic of Korea); ESSW = east surface seawater collected around Nagok-ri (Uljin, Republic of Korea); ESGW = east saline groundwater collected around Hoojeong-ri (Uljin, Republic of Korea); GAPDH = glyceraldehyde 3-phosphate dehydrogenase. ^a^ *p* < 0.01 as compared with intact control by MW test; ^b^ *p* < 0.01 and ^c^ *p* < 0.05 as compared with DNCB control by MW test.

**Table 7:** Changes on the skin antioxidant defense systems after 6 weeks of continuous bathing on seawaters or topical application of DEXA in DNCB-induced AD mice.

Groups	Skin antioxidant defense systems		
	Glutathione (*μ*M/mg of protein)	Lipid peroxidation-malondialdehyde (nM/mg of protein)	Superoxide anion production (NBT reduction/OD at 600 nm)
Controls			
Intact	1.49 ± 0.27	0.38 ± 0.13	0.41 ± 0.12
DNCB	0.44 ± 0.17^a^	2.66 ± 0.70^d^	1.68 ± 0.27^d^
Reference			
DEXA	0.43 ± 0.13^a^	2.58 ± 0.87^d^	1.57 ± 0.62^d^
Bathing on			
WSSW	0.67 ± 0.10^ac^	1.83 ± 0.35^df^	1.28 ± 0.26^df^
WSGW	0.83 ± 0.22^ab^	1.38 ± 0.22^de^	1.03 ± 0.22^de^
ESSW	0.76 ± 0.18^ab^	1.72 ± 0.20^df^	1.14 ± 0.17^de^
ESGW	1.01 ± 0.27^ab^	1.03 ± 0.31^de^	0.73 ± 0.25^de^

Values are expressed as mean ± SD of eight hairless mice. AD = allergic/atopic-like dermatitis; DNCB = 2,4-dinitrochlorobenzene; DEXA = dexamethasone-water soluble; WSSW = west surface seawater collected around Wepo-ri (Ganghwa-do, Republic of Korea); WSGW = west saline groundwater collected at Yonggungoncheon (Seokmo-do, Republic of Korea); ESSW = east surface seawater collected around Nagok-ri (Uljin, Republic of Korea); ESGW = east saline groundwater collected around Hoojeong-ri (Uljin, Republic of Korea); OD = optical density; NBT = nitro blue tetrazolium; LSD = least-significant differences multicomparison. ^a^ *p* < 0.01 as compared with intact control by LSD test; ^b^ *p* < 0.01 and ^c^ *p* < 0.05 as compared with DNCB control by LSD test; ^d^ *p* < 0.01 as compared with intact control by MW test; ^e^ *p* < 0.01 and ^f^ *p* < 0.05 as compared with DNCB control by MW test.

**Table 8:** General skin tissues histomorphometry after 6 weeks of continuous bathing on seawaters or topical application of DEXA in DNCB-induced AD mice.

Index	Controls		DEXA	Bathing on			
	Intact	DNCB		WSSW	WSGW	ESSW	ESGW
Epithelial Th	29.72 ± 6.61	113.81 ± 21.68^d^	36.60 ± 5.03^ef^	70.08 ± 15.31^df^	53.28 ± 10.49^df^	59.34 ± 11.17^df^	46.95 ± 7.80^df^
Collagen fOP	41.20 ± 6.97	79.36 ± 7.91^a^	78.48 ± 7.72^a^	65.35 ± 9.43^ac^	58.92 ± 8.01^ac^	62.00 ± 8.66^ac^	51.79 ± 7.77^bc^
Infiltrated cells							
IF	22.00 ± 11.54	390.25 ± 115.56^d^	70.13 ± 21.72^df^	233.38 ± 60.46^df^	161.63 ± 26.80^df^	215.75 ± 18.91^df^	126.25 ± 29.03^df^
Mast	124.88 ± 42.04	819.25 ± 102.87^a^	221.50 ± 37.03^bc^	628.25 ± 101.19^ac^	492.88 ± 112.89^ac^	572.50 ± 92.16^ac^	341.00 ± 71.43^ac^

Values are expressed as mean ± SD of eight hairless mice. AD = allergic/atopic-like dermatitis; DNCB = 2,4-dinitrochlorobenzene; DEXA = dexamethasone-water soluble; WSSW = west surface seawater collected around Wepo-ri (Ganghwa-do, Republic of Korea); WSGW = west saline groundwater collected at Yonggungoncheon (Seokmo-do, Republic of Korea); ESSW = east surface seawater collected around Nagok-ri (Uljin, Republic of Korea); ESGW = east saline groundwater collected around Hoojeong-ri (Uljin, Republic of Korea); Th = thickness; fOP = fiber occupied region percentages; IF = inflammatory. ^a^ *p* < 0.01 and ^b^ *p* < 0.05 as compared with intact control by LSD test; ^c^ *p* < 0.01 as compared with DNCB control by LSD test; ^d^ *p* < 0.01 and ^e^ *p* < 0.05 as compared with intact control by MW test; ^f^ *p* < 0.01 as compared with DNCB control by MW test.

**Table 9:** Immunohistochemical skin tissues histomorphometry after 6 weeks of continuous bathing on seawaters or topical application of DEXA in DNCB-induced AD mice.

Antiserum	Controls		DEXA	Bathing on			
	Intact	DNCB		WSSW	WSGW	ESSW	ESGW
Caspase-3	3.13 ± 1.96	85.00 ± 7.33^a^	37.00 ± 10.62^ab^	71.13 ± 7.04^ab^	54.25 ± 10.62^ab^	64.13 ± 11.47^ab^	34.13 ± 10.74^ab^
PARP	6.50 ± 2.20	85.00 ± 10.01^c^	21.38 ± 5.40^cd^	69.25 ± 9.47^ce^	48.00 ± 14.58^cd^	55.13 ± 11.29^cd^	38.63 ± 12.32^cd^
Nitrotyrosine	16.38 ± 3.74	87.63 ± 6.55^c^	16.63 ± 3.50^d^	71.38 ± 10.93^cd^	42.50 ± 10.63^cd^	60.75 ± 12.38^cd^	27.25 ± 7.27^cd^
4-HNE	3.63 ± 1.41	85.38 ± 12.78^c^	24.25 ± 7.03^cd^	67.50 ± 6.12^ce^	55.38 ± 11.12^cd^	64.13 ± 11.78^cd^	39.63 ± 15.05^cd^
MMP-9	10.37 ± 5.62	55.34 ± 10.24^a^	38.74 ± 10.80^ab^	41.53 ± 6.54^ab^	34.69 ± 7.06^ab^	39.28 ± 7.70^ab^	24.68 ± 6.22^ab^
IFN-*γ*	7.42 ± 2.04	138.52 ± 25.95^c^	39.46 ± 15.48^cd^	110.67 ± 13.62^ce^	83.71 ± 13.67^cd^	101.45 ± 12.69^cd^	61.28 ± 12.98^cd^
iNOS	6.68 ± 2.14	237.05 ± 28.70^c^	62.15 ± 20.37^cd^	196.20 ± 23.12^ce^	133.28 ± 39.82^cd^	154.53 ± 20.51^cd^	88.08 ± 19.61^cd^
IL-1*β*	4.83 ± 2.37	185.70 ± 26.59^c^	41.73 ± 14.26^cd^	129.77 ± 31.57^cd^	87.36 ± 13.42^cd^	110.18 ± 11.01^cd^	62.02 ± 17.18^cd^
IL-2	3.52 ± 1.75	86.46 ± 20.19^c^	17.15 ± 6.12^cd^	45.13 ± 9.42^cd^	30.47 ± 11.94^cd^	39.48 ± 17.42^cd^	23.50 ± 5.76^cd^
TNF-*α*	5.22 ± 1.29	65.15 ± 14.79^c^	16.42 ± 5.19^cd^	38.96 ± 14.48^cd^	24.26 ± 3.42^cd^	32.67 ± 9.78^cd^	20.97 ± 2.58^cd^

Values are expressed as mean ± SD of eight hairless mice. AD = allergic/atopic-like dermatitis; DNCB = 2,4-dinitrochlorobenzene; DEXA = dexamethasone-water soluble; WSSW = west surface seawater collected around Wepo-ri (Ganghwa-do, Republic of Korea); WSGW = west saline groundwater collected at Yonggungoncheon (Seokmo-do, Republic of Korea); ESSW = east surface seawater collected around Nagok-ri (Uljin, Republic of Korea); ESGW = east saline groundwater collected around Hoojeong-ri (Uljin, Republic of Korea); PARP = cleaved poly (ADP-ribose) polymerase; 4-HNE = 4-hydroxynonenal; MMP = matrix metalloprotease; IFN = interferon; IL = interleukin; iNOS = inducible nitric oxide synthase (2); TNF = tumor necrosis factor. ^a^ *p* < 0.01 as compared with intact control by LSD test; ^b^ *p* < 0.01 as compared with DNCB control by LSD test; ^c^ *p* < 0.01 as compared with intact control by MW test; ^d^ *p* < 0.01 and ^e^ *p* < 0.05 as compared with DNCB control by MW test.

**Table 10:** Splenic tissues histomorphometry after 6 weeks of continuous bathing on seawaters or topical application of DEXA in DNCB-induced AD mice.

Index	Controls		DEXA	Bathing on			
	Intact	DNCB		WSSW	WSGW	ESSW	ESGW
Total Th	1.55 ± 0.28	3.42 ± 0.59^a^	1.67 ± 0.29^c^	2.34 ± 0.39^ac^	1.92 ± 0.25^bc^	2.08 ± 0.25^ac^	1.73 ± 0.12^c^
White pulp	12.75 ± 1.49	14.00 ± 1.69	12.88 ± 2.03	14.38 ± 2.13	13.00 ± 1.51	13.25 ± 1.67	13.63 ± 2.72
Red pulp cells	2.91 ± 0.87	29.40 ± 7.33^a^	5.09 ± 2.65^bc^	21.83 ± 3.20^ad^	15.54 ± 2.15^ac^	18.37 ± 2.19^ac^	11.21 ± 4.27^ac^
Immunoreactive cells							
TNF-*α*	14.13 ± 6.20	430.88 ± 90.74^a^	46.50 ± 19.67^ac^	287.38 ± 79.22^ac^	162.25 ± 39.33^ac^	220.75 ± 56.24^ac^	96.63 ± 16.40^ac^
IFN-*γ*	57.13 ± 28.54	697.63 ± 121.01^a^	132.75 ± 39.64^ac^	526.25 ± 67.50^ad^	360.00 ± 100.05^ac^	413.88 ± 87.74^ac^	239.13 ± 81.36^ac^
iNOS	64.38 ± 20.28	791.25 ± 144788	146.63 ± 30.65^ac^	574.00 ± 117.85^ac^	385.50 ± 72.73^ac^	495.38 ± 81.59^ac^	286.63 ± 82.41^ac^
IL-1*β*	143.38 ± 49.11	814.88 ± 172.80^a^	145.63 ± 65.53^c^	607.63 ± 105.33^ad^	415.38 ± 114.67^ac^	551.88 ± 111.26^ac^	332.88 ± 98.48^ac^
IL-2	89.38 ± 47.11	889.88 ± 148.36^a^	138.88 ± 45.04^bc^	594.75 ± 141.99^ac^	367.13 ± 107.45^ac^	462.38 ± 117.65^ac^	240.88 ± 117.08^ac^

Values are expressed as mean ± SD of eight hairless mice. AD = allergic/atopic-like dermatitis; DNCB = 2,4-dinitrochlorobenzene; DEXA = dexamethasone-water soluble; WSSW = west surface seawater collected around Wepo-ri (Ganghwa-do, Republic of Korea); WSGW = west saline groundwater collected at Yonggungoncheon (Seokmo-do, Republic of Korea); ESSW = east surface seawater collected around Nagok-ri (Uljin, Republic of Korea); ESGW = east saline groundwater collected around Hoojeong-ri (Uljin, Republic of Korea); Th = thickness; IFN = interferon; IL = interleukin; iNOS = inducible nitric oxide synthase (2); TNF = tumor necrosis factor. ^a^ *p* < 0.01 and ^b^ *p* < 0.05 as compared with intact control by MW test; ^c^ *p* < 0.01 and ^d^ *p* < 0.05 as compared with DNCB control by MW test.

**Table 11:** Submandibular LN tissues histomorphometry after 6 weeks of continuous bathing on seawaters or topical application of DEXA in DNCB-induced AD mice.

Index	Controls		DEXA	Bathing on			
	Intact	DNCB		WSSW	WSGW	ESSW	ESGW
Total Th	0.73 ± 0.17	1.70 ± 0.39^a^	0.98 ± 0.20^bc^	1.34 ± 0.14^ac^	1.09 ± 0.17^ac^	1.28 ± 0.22^ac^	0.97 ± 0.12^bc^
Follicles	11.38 ± 2.77	35.00 ± 5.81^a^	14.50 ± 2.73^c^	28.25 ± 5.60^ac^	22.88 ± 3.60^ac^	25.25 ± 5.75^ac^	14.63 ± 2.07^ac^
Cortex Th	353.27 ± 104.03	981.34 ± 123.88^a^	399.76 ± 98.96^c^	822.82 ± 100.84^ac^	753.92 ± 123.13^ac^	807.70 ± 119.32^ac^	528.73 ± 128.20^ac^
Immunoreactive cells							
TNF-*α*	7.52 ± 2.03	131.36 ± 24.32^d^	20.13 ± 11.80^de^	94.60 ± 12.60^de^	65.21 ± 23.35^de^	91.63 ± 14.44^de^	38.52 ± 11.13^de^
IFN-*γ*	7.80 ± 5.27	206.72 ± 15.82^d^	18.97 ± 5.99^de^	157.95 ± 25.35^de^	79.41 ± 15.14^de^	112.41 ± 14.85^de^	46.65 ± 27.73^de^
iNOS	8.65 ± 4.11	153.55 ± 24.80^d^	19.38 ± 7.03^de^	113.57 ± 21.64^de^	64.26 ± 12.58^de^	82.10 ± 26.17^de^	37.52 ± 15.17^de^
IL-1*β*	15.07 ± 6.75	123.60 ± 20.31^d^	43.47 ± 12.23^de^	94.51 ± 13.83^de^	63.23 ± 95.66^de^	78.81 ± 18.19^de^	42.50 ± 14.74^de^
IL-2	2.85 ± 1.54	84.57 ± 17.53^d^	15.30 ± 5.39^de^	45.56 ± 13.55^de^	29.20 ± 7.20^de^	31.96 ± 8.98^de^	21.36 ± 72.07^de^

Values are expressed as mean ± SD of eight hairless mice. AD = allergic/atopic-like dermatitis; DNCB = 2,4-dinitrochlorobenzene; DEXA = dexamethasone-water soluble; WSSW = west surface seawater collected around Wepo-ri (Ganghwa-do, Republic of Korea); WSGW = west saline groundwater collected at Yonggungoncheon (Seokmo-do, Republic of Korea); ESSW = east surface seawater collected around Nagok-ri (Uljin, Republic of Korea); ESGW = east saline groundwater collected around Hoojeong-ri (Uljin, Republic of Korea); LN = lymph node; Th = thickness; IFN = interferon; IL = interleukin; iNOS = inducible nitric oxide synthase (2); TNF = tumor necrosis factor. ^a^ *p* < 0.01 and ^b^ *p* < 0.05 as compared with intact control by LSD test; ^c^ *p* < 0.01 as compared with DNCB control by LSD test; ^d^ *p* < 0.01 as compared with intact control by MW test; ^e^ *p* < 0.01 as compared with DNCB control by MW test.

## References

[1] Li C., Lasse S., Lee P. (2010). Development of atopic dermatitis-like skin disease from the chronic loss of epidermal caspase-8. *Proceedings of the National Academy of Sciences of the United States of America*.

[2] Leung D. Y. M. (2000). Atopic dermatitis: new insights and opportunities for therapeutic intervention. *The Journal of Allergy and Clinical Immunology*.

[3] Mar W. C., Lee S.-J., Nam K.-W. (2011). Essential oil of *Thujopsis dolobrata* suppresses atopic dermatitis-like skin lesions in NC/Nga mice. *Biomolecules & Therapeutics*.

[4] Park G., Oh M. S. (2014). Inhibitory effects of *Juglans mandshurica* leaf on allergic dermatitis-like skin lesions-induced by 2,4-dinitrochlorobenzene in mice. *Experimental and Toxicologic Pathology*.

[5] Pugliarello S., Cozzi A., Gisondi P., Girolomoni G. (2011). Phenotypes of atopic dermatitis. *Journal der Deutschen Dermatologischen Gesellschaft*.

[6] Kim S. R., Choi H.-S., Seo H. S., Choi Y. K., Shin Y. C., Ko S.-G. (2012). Topical application of herbal mixture extract inhibits ovalbumin- or 2,4-dinitrochlorobenzene-induced atopic dermatitis. *Evidence-Based Complementary and Alternative Medicine*.

[7] Koga C., Kabashima K., Shiraishi N., Kobayashi M., Tokura Y. (2008). Possible pathogenic role of Th17 cells for atopic dermatitis. *The Journal of Investigative Dermatology*.

[8] Tasaka K. (1986). Anti-allergic drugs. *Drugs of Today*.

[9] Merial-Kieny C., Mengual X., Guerrero D., Sibaud V. (2011). Clinical efficacy of Avène hydrotherapy measured in a large cohort of more than 10,000 atopic or psoriatic patients. *Journal of the European Academy of Dermatology and Venereology*.

[10] Léauté-Labrèze C., Saillour F., Chêne G. (2001). Saline spa water or combined water and UV-B for psoriasis vs conventional UV-B: lessons from the Salies de Béarn randomized study. *Archives of Dermatology*.

[11] Tsoureli-Nikita E., Menchini G., Ghersetich I., Hercogova J. (2002). Alternative treatment of psoriasis with balneotherapy using Leopoldine spa water. *Journal of the European Academy of Dermatology and Venereology*.

[12] Brockow T., Schiener R., Franke A., Resch K. L., Peter R. U. (2007). A pragmatic randomized controlled trial on the effectiveness of low concentrated saline spa water baths followed by ultraviolet B (UVB) compared to UVB only in moderate to severe psoriasis. *Journal of the European Academy of Dermatology and Venereology*.

[13] Peroni A., Gisondi P., Zanoni M., Girolomoni G. (2008). Balneotherapy for chronic plaque psoriasis at Comano spa in Trentino, Italy. *Dermatologic Therapy*.

[14] Portalès P., Ariès M.-F., Licu D. (2001). Immunomodulation induced by Avène spring water on Th1- and Th2-dependent cytokine production in healthy subjects and atopic dermatitis patients. *Skin Pharmacology and Applied Skin Physiology*.

[15] Fioravanti A., Lamboglia A., Pascarelli N. A. (2013). Thermal water of Vetriolo, Trentino, inhibits the negative effect of interleukin-1*β* on nitric oxide production and apoptosis in human osteoarthritic chondrocyte. *Journal of Biological Regulators and Homeostatic Agents*.

[16] Prandelli C., Parola C., Buizza L. (2013). Sulphurous thermal water increases the release of the anti-inflammatory cytokine IL-10 and modulates antioxidant enzyme activity. *International Journal of Immunopathology and Pharmacology*.

[17] Soria M., González-Haro C., Esteva S., Escanero J. F., Pina J. R. (2014). Effect of sulphurous mineral water in haematological and biochemical markers of muscle damage after an endurance exercise in well-trained athletes. *Journal of Sports Sciences*.

[18] Yoon Y. S., Sajo M. E., Ignacio R. M., Kim S., Kim C., Lee K. (2014). Positive Effects of hydrogen water on 2,4-dinitrochlorobenzene-induced atopic dermatitis in NC/Nga mice. *Biological & Pharmaceutical Bulletin*.

[19] Hataguchi Y., Tai H., Nakajima H., Kimata H. (2005). Drinking deep-sea water restores mineral imbalance in atopic eczema/dermatitis syndrome. *European Journal of Clinical Nutrition*.

[20] Adler-Cohen C., Czarnowicki T., Dreiher J., Ruzicka T., Ingber A., Harari M. (2012). Climatotherapy at the Dead Sea: an effective treatment modality for atopic dermatitis with significant positive impact on quality of life. *Dermatitis*.

[21] Bak J.-P., Kim Y.-M., Son J., Kim C.-J., Kim E.-H. (2012). Application of concentrated deep sea water inhibits the development of atopic dermatitis-like skin lesions in NC/Nga mice. *BMC Complementary and Alternative Medicine*.

[22] Kang G.-J., Han S.-C., Yi E.-J., Kang H.-K., Yoo E.-S. (2011). The inhibitory effect of premature *Citrus unshiu* extract on atopic dermatitis in vitro and in vivo. *Toxicological Research*.

[23] Park M. S., Chang B. S., Kim D. H. (2011). The bathing effect of mineral-rich water on atopic dermatitis like skin lesions in hairless mice. *Journal of Investigative Cosmetology*.

[24] Park M.-Y., Choi H.-Y., Kim J.-D., Lee H.-S., Ku S.-K. (2010). 28 Days repeated oral dose toxicity test of aqueous extracts of mahwangyounpae-tang, a polyherbal formula. *Food and Chemical Toxicology*.

[25] Yoon H. S., Kim J. W., Cho H. R. (2010). Immunomodulatory effects of Aureobasidium pullulans SM-2001 exopolymers on the cyclophosphamide-treated mice. *Journal of Microbiology and Biotechnology*.

[26] Clark B. D., Bedrosian I., Schindler R. (1991). Detection of interleukin 1*α* and 1*β* in rabbit tissues during endotoxemia using sensitive radioimmunoassays. *Journal of Applied Physiology*.

[27] Kim K. H., Park S. J., Lee Y. J. (2015). Inhibition of UVB-induced skin damage by exopolymers from *Aureobasidium pullulans* SM-2001 in hairless mice. *Basic & Clinical Pharmacology & Toxicology*.

[28] Campanini M. Z., Pinho-Ribeiro F. A., Ivan A. L. M. (2013). Efficacy of topical formulations containing *Pimenta pseudocaryophyllus* extract against UVB-induced oxidative stress and inflammation in hairless mice. *Journal of Photochemistry and Photobiology B: Biology*.

[29] Lowry O. H., Rosebrough N. J., Farr A. L., Randall R. J. (1951). Protein measurement with the Folin phenol reagent. *The Journal of Biological Chemistry*.

[30] Jamall I. S., Smith J. C. (1985). Effects of cadmium on glutathione peroxidase, superoxide dismutase, and lipid peroxidation in the rat heart: a possible mechanism of cadmium cardiotoxicity. *Toxicology and Applied Pharmacology*.

[31] Barbosa D. S., Cecchini R., El Kadri M. Z., Rodríguez M. A. M., Burini R. C., Dichi I. (2003). Decreased oxidative stress in patients with ulcerative colitis supplemented with fish oil *ω*-3 fatty acids. *Nutrition*.

[32] Harrigan T. J., Abdullaev I. F., Jourd'heuil D., Mongin A. A. (2008). Activation of microglia with zymosan promotes excitatory amino acid release via volume-regulated anion channels: the role of NADPH oxidases. *Journal of Neurochemistry*.

[33] Ryu J. C., Park S. M., Hwangbo M. (2013). Methanol extract of artemisia apiacea hance attenuates the expression of inflammatory mediators via NF- B inactivation. *Evidence-Based Complementary and Alternative Medicine*.

[34] Yang J. H., Kim S. C., Shin B. Y. (2013). O-methylated flavonol isorhamnetin prevents acute inflammation through blocking of NF-*κ*B activation. *Food and Chemical Toxicology*.

[35] Kang S. J., Lee J. E., Lee E. K. (2014). Fermentation with *Aquilariae Lignum* enhances the anti-diabetic activity of green tea in type II diabetic db/db mouse. *Nutrients*.

[36] Im L.-R., Ahn J.-Y., Kim J.-H. (2011). Inhibitory effect of Kyungohkgo in the development of 2,4-dinitrochlorobenzene-induced atopic dermatitis in NC/Nga mice. *Archives of Pharmacal Research*.

[37] Nystad W., Røysamb E., Magnus P., Tambs K., Harris J. R. (2005). A comparison of genetic and environmental variance structures for asthma, hay fever and eczema with symptoms of the same diseases: a study of Norwegian twins. *International Journal of Epidemiology*.

[38] Nadworny P. L., Wang J., Tredget E. E., Burrell R. E. (2008). Anti-inflammatory activity of nanocrystalline silver in a porcine contact dermatitis model. *Nanomedicine: Nanotechnology, Biology, and Medicine*.

[39] Stone K. D., Prussin C., Metcalfe D. D. (2010). IgE, mast cells, basophils, and eosinophils. *The Journal of Allergy and Clinical Immunology*.

[40] Choi J. H., Kim H. G., Jin S. W. (2013). Topical application of *Pleurotus eryngii* extracts inhibits 2,4-dinitrochlorobenzene-induced atopic dermatitis in NC/Nga mice by the regulation of Th1/Th2 balance. *Food and Chemical Toxicology*.

[41] Sawada E., Yoshida N., Sugiura A., Imokawa G. (2012). Th1 cytokines accentuate but Th2 cytokines attenuate ceramide production in the stratum corneum of human epidermal equivalents: an implication for the disrupted barrier mechanism in atopic dermatitis. *Journal of Dermatological Science*.

[42] Vestergaard C., Yoneyama H., Murai M. (1999). Overproduction of Th2-specific chemokines in NC/Nga mice exhibiting atopic dermatitis-like lesions. *The Journal of Clinical Investigation*.

[43] Poulsen L. K., Hummelshoj L. (2007). Triggers of IgE class switching and allergy development. *Annals of Medicine*.

[44] Yamanaka K.-I., Mizutani H. (2011). The role of cytokines/chemokines in the pathogenesis of atopic dermatitis. *Current Problems in Dermatology*.

[45] Whittle B. J. R., Varga C., Berko A. (2008). Attenuation of inflammation and cytokine production in rat colitis by a novel selective inhibitor of leukotriene A_4_ hydrolase. *The British Journal of Pharmacology*.

[46] Schottelius A. J., Zügel U., Döcke W.-D. (2010). The role of mitogen-activated protein kinase-activated protein kinase 2 in the p38/TNF-*α* pathway of systemic and cutaneous inflammation. *The Journal of Investigative Dermatology*.

[47] Smith K. A. (1988). Interleukin-2: inception, impact, and implications. *Science*.

[48] Fallahzadeh M. K., Roozbeh J., Geramizadeh B., Namazi M. R. (2011). Interleukin-2 serum levels are elevated in patients with uremic pruritus: a novel finding with practical implications. *Nephrology Dialysis Transplantation*.

[49] Isaacs A., Tizard I. (1995). Lymphokines and cytokines. *Immunology: An introduction*.

[50] Ku H.-O., Jeong S.-H., Kang H.-G. (2008). Intracellular expression of cytokines and granzyme B in auricular lymph nodes draining skin exposed to irritants and sensitizers. *Toxicology*.

[51] Szabó C. (1995). Alterations in nitric oxide production in various forms of circulatory shock. *New Horizons: Science and Practice of Acute Medicine*.

[52] Lee C. S., Yi E. H., Kim H.-R. (2011). Anti-dermatitis effects of oak wood vinegar on the DNCB-induced contact hypersensitivity via STAT3 suppression. *Journal of Ethnopharmacology*.

[53] Lee J. K., Park J. H., Park S. H., Kim H. S., Oh H. Y. (2002). A nonradioisotopic endpoint for measurement of lymph node cell proliferation in a murine allergic contact dermatitis model, using bromodeoxyuridine immunohistochemistry. *Journal of Pharmacological and Toxicological Methods*.

[54] Sivaranjani N., Rao S. V., Rajeev G. (2013). Role of reactive oxygen species and antioxidants in atopic dermatitis. *Journal of Clinical and Diagnostic Research*.

[55] Odabasoglu F., Cakir A., Suleyman H. (2006). Gastroprotective and antioxidant effects of usnic acid on indomethacin-induced gastric ulcer in rats. *Journal of Ethnopharmacology*.

[56] Pinnell S. R. (2003). Cutaneous photodamage, oxidative stress, and topical antioxidant protection. *Journal of the American Academy of Dermatology*.

[57] Dubinina E. E., Dadali V. A. (2010). Role of 4-hydroxy-trans-2-nonenal in cell functions. *Biochemistry*.

[58] Mohiuddin I., Chai H., Lin P. H., Lumsden A. B., Yao Q., Chen C. (2006). Nitrotyrosine and chlorotyrosine: clinical significance and biological functions in the vascular system. *The Journal of Surgical Research*.

[59] Pacher P., Beckman J. S., Liaudet L. (2007). Nitric oxide and peroxynitrite in health and disease. *Physiological Reviews*.

[60] Cuzzocrea S., Zingarelli B., Hake P., Salzman A. L., Szabó C. (1998). Antiinflammatory effects of mercaptoethylguanidine, a combined inhibitor of nitric oxide synthase and peroxynitrite scavenger, in carrageenan-induced models of inflammation. *Free Radical Biology & Medicine*.

[61] Nuñez G., Benedict M. A., Hu Y., Inohara N. (1998). Caspases: the proteases of the apoptotic pathway. *Oncogene*.

[62] Cole K. K., Perez-Polo J. R. (2002). Poly(ADP-ribose) polymerase inhibition prevents both apoptotic-like delayed neuronal death and necrosis after H_2_O_2_ injury. *Journal of Neurochemistry*.

[63] Sternlicht M. D., Werb Z. (2001). How matrix metalloproteinases regulate cell behavior. *Annual Review of Cell and Developmental Biology*.

[64] Rittié L., Fisher G. J. (2002). UV-light-induced signal cascades and skin aging. *Ageing Research Reviews*.

[65] Devillers A. C. A., Van Toorenenbergen A. W., Klein Heerenbrink G. J., Mulder P. G. H., Oranje A. P. (2007). Elevated levels of plasma matrix metalloproteinase-9 in patients with atopic dermatitis: a pilot study. *Clinical and Experimental Dermatology*.

[66] Lü Z.-R., Park D., Lee K.-A. (2009). Profiling the dysregulated genes of keratinocytes in atopic dermatitis patients: cDNA microarray and interactomic analyses. *The Journal of Dermatological Science*.

[67] Harper J. I., Godwin H., Green A. (2010). A study of matrix metalloproteinase expression and activity in atopic dermatitis using a novel skin wash sampling assay for functional biomarker analysis. *The British Journal of Dermatology*.

